# Chromosomal Gene Movements Reflect the Recent Origin and Biology of Therian Sex Chromosomes 

**DOI:** 10.1371/journal.pbio.0060080

**Published:** 2008-04-01

**Authors:** Lukasz Potrzebowski, Nicolas Vinckenbosch, Ana Claudia Marques, Frédéric Chalmel, Bernard Jégou, Henrik Kaessmann

**Affiliations:** 1 Center for Integrative Genomics, University of Lausanne, Lausanne, Switzerland; 2 INSERM U625, IFR 140, Université Rennes I, Campus de Beaulieu, Rennes, France; Université Claude Bernard, France

## Abstract

Mammalian sex chromosomes stem from ancestral autosomes and have substantially differentiated. It was shown that X-linked genes have generated duplicate intronless gene copies (retrogenes) on autosomes due to this differentiation. However, the precise driving forces for this out-of-X gene “movement” and its evolutionary onset are not known. Based on expression analyses of male germ-cell populations, we here substantiate and extend the hypothesis that autosomal retrogenes functionally compensate for the silencing of their X-linked housekeeping parental genes during, but also after, male meiotic sex chromosome inactivation (MSCI). Thus, sexually antagonistic forces have not played a major role for the selective fixation of X-derived gene copies in mammals. Our dating analyses reveal that although retrogenes were produced ever since the common mammalian ancestor, selectively driven retrogene export from the X only started later, on the placental mammal (eutherian) and marsupial (metatherian) lineages, respectively. Together, these observations suggest that chromosome-wide MSCI emerged close to the eutherian–marsupial split approximately 180 million years ago. Given that MSCI probably reflects the spread of the recombination barrier between the X and Y, crucial for their differentiation, our data imply that these chromosomes became more widely differentiated only late in the therian ancestor, well after the divergence of the monotreme lineage. Thus, our study also provides strong independent support for the recent notion that our sex chromosomes emerged, not in the common ancestor of all mammals, but rather in the therian ancestor, and therefore are much younger than previously thought.

## Author Summary

Our sex chromosomes have profoundly differentiated since evolving from an ancestral pair of non-sex chromosomes (autosomes). In this study, we first show that X chromosome–derived retrogenes (genes that arose as duplicates of “parental” X-linked genes) are specifically expressed during the meiotic and postmeiotic stages of spermatogenesis, thus functionally replacing their parents during, but also after, the process of male meiotic sex chromosome inactivation (MSCI). We then show that the “export” of retroposed gene copies from the X chromosome started rather recently during mammalian evolution, on the eutherian (“placental” mammal) and marsupial lineages, respectively. This suggests that MSCI—the main driving force for this out of the X gene “movement”—originated around the separation of these two major (therian) mammalian lineages, approximately 180 million years ago. Given that MSCI was likely triggered as soon as the proto-X and -Y chromosomes ceased to recombine (an event that marks the origin of these sex chromosomes), our data also support the recent notion that our sex chromosomes and those of other therians emerged, not in the common ancestor of all mammals, but—probably rather late—in the therian ancestor.

## Abstract 3

By dating the evolutionary onset of gene export from the X chromosome, researchers reveal the age of meiotic sex chromosome inactivation in mammals and suggest a rather recent origin of our sex chromosomes.

## Introduction

Several recent studies [1–3] of mammalian retroduplicate genes (i.e., intronless duplicate genes generated by the reverse transcription of mRNAs from “parental” source genes [4,5]) have revealed a peculiar pattern with respect to their chromosomal origin: an excess of functional retrogenes stem from the X chromosome. It was suggested that these autosomal retroduplicate counterparts of X-linked genes carry out functions of the silenced parental genes that are necessary or advantageous during the transcriptional silencing of the X chromosome in the meiotic phase of spermatogenesis (termed male meiotic sex chromosome inactivation [MSCI] [6]), and were therefore selectively fixed during evolution [1,7]. In support of this notion, a number of X-derived retrogenes were found to be expressed in testis [1–3,7], and for some retrogenes, it was shown that they are expressed during meiosis while their parental genes are shut off (e.g., [8]). In addition, loss of function of two X-derived retrogenes was shown to lead to severe defects of male meiotic functions in humans and mice [9–11], suggesting that such genes are needed to replace their parental genes during male meiosis.

When did the selectively driven, out-of-X movement of genes begin? If MSCI is responsible for export of gene copies from the X, answering this question would provide a unique means to date the evolutionary onset of MSCI.

## Results and Discussion

### An Excess of X-Derived Retrogenes in Eutherian and Marsupial Genomes

To trace the evolution of gene movements in mammals, we first screened for intronless retroposed gene copies (retrocopies) and their parental genes in three eutherian (“placental” mammal) genomes and one metatherian (“marsupial”) genome (opossum), using a refinement of our previously described procedure [2] (Materials and Methods). This analysis identified several thousand retrocopies in each of the therian genomes analyzed (Table 1). Thus, the process of retroposition has significantly shaped, not only the genomic landscape of eutherians [1,2], but also that of its sister lineage, the marsupials.

**Table 1:**
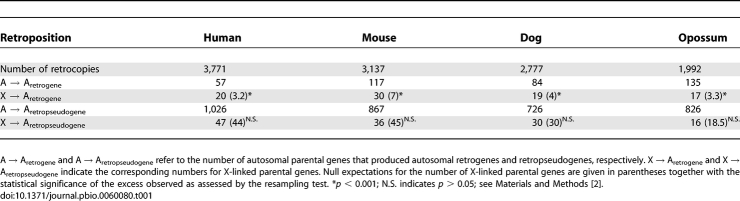
Retroposition in Therian Genomes

We then extracted two subsets from these retrocopy data for each species (see Materials and Methods for details): One was enriched for functional retrocopies (retrogenes; Tables 1 and S1–S4), whereas the other contained retropseudogenes with open reading frame disruptions (premature stop codons and frameshifts) that likely preclude gene function (Table 1).

The analysis of chromosomal locations of parental genes revealed that X-linked genes of all genomes analyzed have spawned a large excess of functional retrogenes compared to autosomal genes, whereas no such bias is observed for parental genes that gave rise to retropseudogenes (Table 1). Thus, preferential fixation of functional X-derived genes by natural selection occurred, not only in eutherians [1,2], but also in metatherians. The latter is consistent with a recent study that showed that MSCI occurs in marsupials [12].

### Autosomal Retrogenes Compensate for the Transcriptional Silencing of Their X-Linked Parental Genes

**Figure 1:**
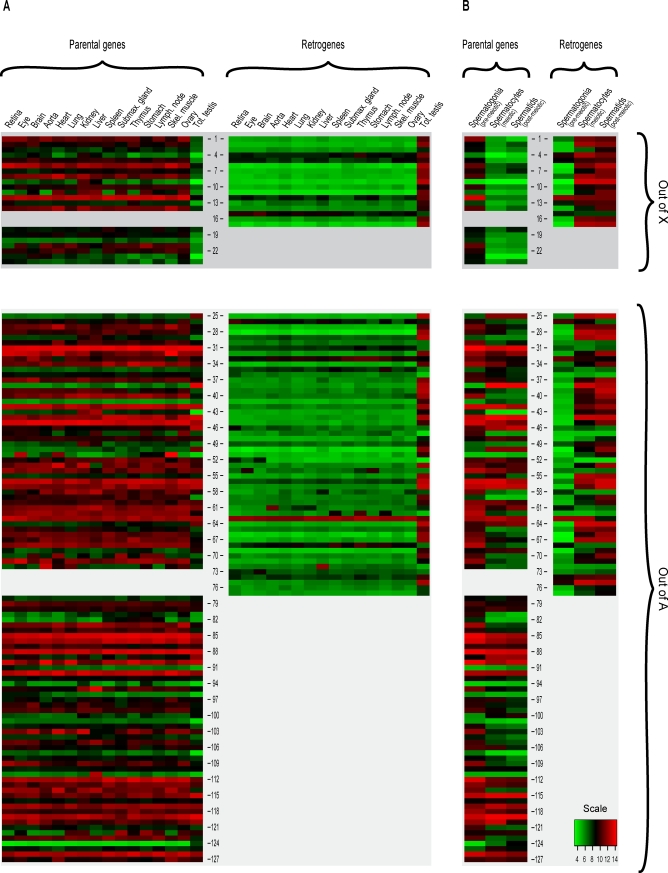
Expression Pattern of Mouse Parental Genes and Retrogenes in Somatic and Germline Tissues (A), and during Spermatogenesis (B) Log2 expression signals are represented according to the plotted scale. The two heat maps show signal values for both parental genes (left side) and their respective retrogenes (right side), or only for one of the two, when data are not available for the other. Upper and lower parts of the panels contain pairs with parental genes located on the X chromosome and the autosomes, respectively. Line numbers in the middle of the heat maps correspond to mouse retrogene identifiers in Tables S2 and S5.

Before examining the evolutionary history of X movement patterns in more detail, we sought to obtain further evidence for the hypothesis that MSCI is the driving force for the preferential copying of genes from the X to autosomes, which was so far based on the analysis of individual genes (see Introduction). To this end, we analyzed expression patterns of retrogenes and their parental genes using genome-wide murine expression data [13] from testicular germ-cell populations, total testis, ovary, and 14 somatic tissues (Figure 1A; Materials and Methods).

We find that all parental genes are broadly expressed (median: 16, mean: ∼14.2 tissues), in significantly more tissues than other genes in the genome (median: 15, mean: ∼11 tissues, *p* < 10^−11^, Mann-Whitney *U* test; Figure 1A), which substantiates previous notions that retrogenes stem from housekeeping genes with important functions in all or most tissues [2,7]. In contrast, the majority of X-derived retrogenes (12 of 17, ∼71%) are specifically expressed in testes (Figure 1A and Table S5). X-derived retrogenes show a striking excess of testis-specific cases compared to their parental genes (0 of 21 specifically expressed in testes) or other genes in the genome (790 of 14,991, 5.3%; *p* < 10^−17^, Fisher exact test). We note that similar patterns have been described in *Drosophila* [14], a genus in which the out-of-X movement of genes was originally observed [15]. X-derived retrogenes in our data are also significantly more frequently expressed (specifically or nonspecifically) in testis (17 of 17, or 100%, with testis expression) compared to other, autosome-derived retrogenes (41 of 53, or ∼77%, with testis expression; two-tailed *p* < 0.05, Fisher exact test; 26 of 53, or 49%, are testis-specific). This points to a selective enrichment of testis functions among X-derived retrogenes during evolution, although retrogenes generally seem to be frequently expressed in testis, consistent with previous studies [2,3].

In order to functionally compensate for their parental genes in testes (Figure 1A), expression of X-derived retrogenes would be specifically required in testicular meiotic germ cells (spermatocytes), where their parental genes are silenced, but not in premeiotic spermatogonia (Figure 1B). Our expression analysis of premeiotic, meiotic, and postmeiotic cells revealed a striking pattern (Figure 1B and Table S5), consistent with a compensation function of retrogenes during but—surprisingly—also after meiosis (see [6] for recent evidence of active postmeiotic silencing of the X). In spermatogonia, X-linked parental genes show high and their retrogene copies low expression activity. Conversely, X-derived retrogenes are highly expressed in spermatocytes and postmeiotic spermatids, while their parental genes are silenced.

The overall propensity of retrogenes—including retrogenes with autosomal progenitors (Figure 1B)—to be expressed in spermatocytes/spermatids is probably due to the “hypertranscription” state of autosomal chromatin in these cell types ([3] and references therein). This likely facilitated the initial transcription of retrocopies after their emergence, allowing them to obtain functions in the late stages of spermatogenesis.

However, X-derived retrogenes are more frequently expressed in spermatocytes than retrogenes with autosomal parental genes (16 of 17, or 94%, X-derived vs. 39 of 53, or 74%, autosome-derived; one-tailed *p* < 0.1, Fisher exact test), and at higher levels (median log2-transformed expression signal: ∼10.9 vs. 8.8, one-tailed *p* < 0.05, Mann-Whitney *U* test). A similar pattern is observed in postmeiotic spermatids (100% vs. 75% expressed, two-tailed *p* < 0.05; median signal: ∼11.0 vs. ∼10.5, one-tailed *p* = 0.15). In addition, based on expression cluster analyses [13] (Materials and Methods), we find that a significant excess of X-derived retrogenes show transcriptional induction in meiosis when compared to retrogenes that stem from autosomes (10 of 17, or ∼59%, vs. 16 of 53, or ∼30%; two-tailed *p* < 0.05, Fisher exact test).

Our expression analyses substantiate the hypothesis that retrogenes that stem from the X have been fixed during evolution and shaped by natural selection to compensate for parental (housekeeping) gene silencing during (and after) MSCI. Thus, sexual antagonism (i.e., evolutionary conflict between males and females), which was previously considered as an alternative driving force for the fixation of X-derived retrogenes [1,16], likely played less significant roles for the selectively driven export of X-linked genes in mammals (at least for those that are specifically expressed during/after meiosis). In contrast to the mammalian pattern, X chromosome inactivation during spermatogenesis does not seem to be a major contributor to the out-of-X movement of genes in *Drosophila* [17]. Rather, it appears that the increased residency time of the X chromosomes in females accounts for the observed pattern in this genus [17]. Thus, interestingly, the predominant selective forces associated with the export of X-linked genes appear to differ between fruitflies (sexually antagonistic selection) and mammals (MSCI).

### Gene Movements Reveal the Evolutionary Onset of Meiotic Sex Chromosome Inactivation

**Figure 2:**
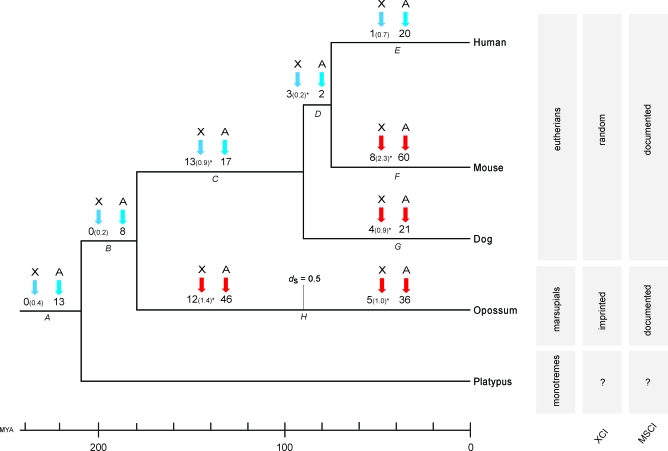
Age of Retrogenes Branches are labeled A to H. “X” refers to the number of X-linked parental genes (in the X conserved region) that produced autosomal retrogenes. “A” refers to the number of autosomal parental genes that generated an autosomal retrogene. The numbers of parental genes producing retrogenes on the lineage leading to humans are indicated by blue arrows; those generating retrogenes on the other lineages are indicated by red arrows. Null expectations for the out-of-X movement (based on the number of genes on the X) are in parentheses, and the significances of resampling tests for observed excesses are indicated (an asterisk [*] indicates *p* < 0.01). The set of opossum-specific retrogenes is separated into two subsets based on *d*_S_ smaller or larger than 0.5, which corresponds to the age of the split of the human–dog lineage (see Materials and Methods). The right side of the figure indicates current knowledge of XCI (female somatic X chromosome inactivation) and MSCI for the major lineages in the tree (see main text for references).

To date the evolutionary onset of the out-of-X movement of genes in mammals, we screened for the presence/absence of human retrogenes in genomes representing the three major mammalian lineages (see Materials and Methods for details). In addition to three eutherian and one marsupial genome (opossum), this analysis included a genome (platypus) of the most basal mammalian lineage, the egg-laying monotremes (Figure 2).

For the purpose of this dating, it is necessary to focus the X-related part of the analysis on the ancestral part of the human X, termed X conserved region [18] (XCR), which is shared across mammals. The dating of human XCR-derived retrogenes uncovered a striking pattern (Figure 2). Although a number of autosomal retrogenes were produced in the common mammalian ancestor more than approximately 210 million years ago (Mya) as well as in the common therian ancestor between approximately 180 and 210 Mya, X-derived genes only started to appear after the eutherian–metatherian split (<180 Mya) on both of the descendent lineages. The approximately 1,300% excess of X-derived retrogenes in the common human–dog ancestor (branch *C*) is highly significant (*p* < 0.01, resampling test), which suggests strong selection driving the fixation of X-derived retrogenes between 90 and 180 Mya on the eutherian lineage. Similarly, there is an approximately 860% excess of old (pairwise *d*_S_ > 0.5 between parental gene and retrogene) marsupial-specific X-derived retrogenes, which suggests selective export of genes from the X early in the metatherian lineage (i.e., early on branch *H*; *p* < 0.01, resampling test). Importantly, the X-to-autosome parental gene ratio is significantly higher on branch *C* (human–dog ancestor) than on branch *B* (common therian ancestor), where the zero observed out-of-X cases correspond to the random expectation (two-tailed *p* < 0.01, Fisher exact test).

These findings demonstrate a significant shift in the selective forces—likely due to the emergence of MSCI—driving genes out of the X around the time of divergence of the two therian lineages. Thus, selective gene export driven by chromosome-wide MSCI originated either just before (not leaving enough time for an X-skew in the retrogene generation pattern on branch *B*) or—less parsimoniously—soon after the eutherian–marsupial split around 180 Mya, which would imply two independent origins of MSCI in eutherians and metatherians, respectively.

### Selective Export of X-Linked Housekeeping Genes upon the Emergence of MSCI

We find that the first described X-derived human retrogene with parental replacement function, *PGK2* ([8]), originated in the common human–dog ancestor approximately 90–180 Mya (Table 2), contrary to a previous study that suggested an origin in the therian ancestor [19]. The *PGK1* parental gene has independently spawned three *PGK* retrogenes on the marsupial lineage (Figure S1A; Table S4, identifiers MD5, MD6, and MD12). One of these marsupial *PGK* genes (Table S4, MD6) shows a high divergence from its parental gene at silent sites (*d*_S_ ∼ 0.77, corresponding to an age of roughly 140 million years), indicating an origin shortly after the eutherian–metatherian split. The *Cetn-2* (*Centrin*) parental gene (a gene required for centromere structure and function [20]) similarly gave rise to retrogenes independently in the human–dog ancestor (Figure S1B and Table 2) and in metatherian evolution (Figure S1B; Table S4, MD9). Both the *PGK* and *Centrin* retrogenes evolved highly specific testis expression patterns in eutherians [8,21] (Figure 1 and Table S5, identifiers MM6 and MM17). These data suggest a strong selective pressure to generate autosomal copies of these important housekeeping genes soon after the evolutionary onset of MSCI in both placental and marsupial mammals.

**Table 2:**
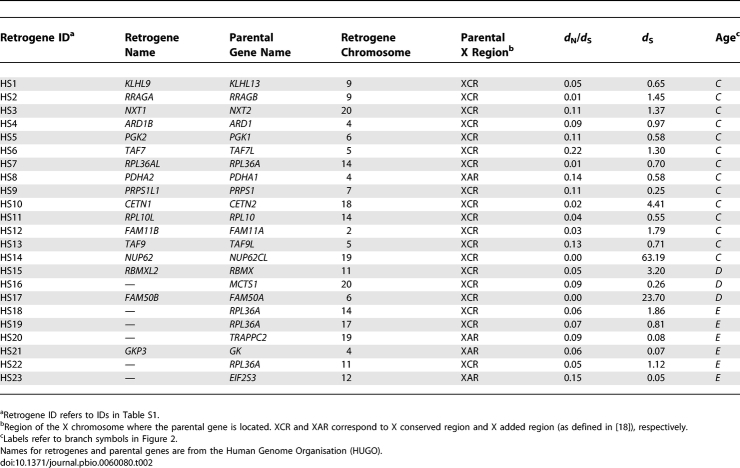
Human X-Derived Retrogenes

Several other parental genes with fundamental cellular functions also spawned functional retrogene copies early in eutherian or metatherian evolution. For example, retrogenes encoding proteins involved in protein synthesis (Table 2, HS7 and HS11), the core transcription machinery (HS6 and HS13), nucleotide synthesis (HS9), and energy metabolism (HS8) originated in the common eutherian ancestor.

### Chromosomal Gene Movements, MSCI, and the Emergence of Therian Sex Chromosomes

Our study on chromosomal gene movements in mammals has general implications for the origin and evolution of mammalian sex chromosomes. The X and Y chromosomes started to evolve from an ancestral autosomal pair when the *SRY* gene—the primary sex determinant—emerged on the proto-Y chromosome in a mammalian ancestor [22,23]. Suppression of recombination between the proto-X and -Y chromosomes initially encompassed the long arm of the X chromosome (containing the *SRY* gene) and then spread to include the entire XCR. This barrier to recombination between the X and Y was crucial for their differentiation and therefore marks the origin of these sex chromosomes [24]. It likely also triggered silencing of genes in the unpaired (nonrecombining) regions of the X during the meiotic phase of spermatogenesis—the process of MSCI—through a more general molecular mechanism (meiotic silencing of unsynapsed chromatin, MSUC) that silences unpaired DNA during meiosis [6,25].

Our study of chromosomal gene movements suggests that MSCI emerged late in the common therian ancestor, around 180 Mya. Intriguingly, given that MSCI likely reflects the spread of the recombination barrier between the X and Y (see above), this observation also suggests that these chromosomes originated after the separation of the therian and monotreme lineages, which is later than the previously suggested origin [22,23], in the common ancestor of all mammals approximately 240–310 Mya.

Our findings are consistent with a recent study of monotreme sex chromosomes [26]. Contrary to previous studies, which suggested that the platypus X chromosomes are related to both the therian X and bird Z chromosomes [27,28], this work only finds homologous relationships between the sex chromosomes of monotremes and birds [26].

A recent origin of X and Y sex chromosomes in therians also implies that all other properties and evolutionary forces associated with the differentiated X and Y chromosomes—such as somatic X chromosome inactivation (XCI) seen in females and sexual antagonism—emerged recently in therians. A recent origin of XCI—which may be derived from MSCI ([25,27])—in the common therian ancestor is consistent with the presence of XCI in eutherians and marsupials, as well as the recent origin of the *XIST* gene, crucial for XCI in eutherians, in the common eutherian ancestor [29,30].

In conclusion, our analyses of gene movement patterns have shed new light on the origin and properties of mammalian sex chromosomes. They suggest that in addition to the well-known phenotypes that distinguish therian mammals from monotremes, such as placentation, which evolved together with viviparity [31], therian mammals have evolved a unique sex chromosome system that includes dosage compensation and MSCI.

## Materials and Methods

### Retrocopy screen.

We identified retrocopies in the human, mouse, dog, and opossum genomes using a previously described procedure [2]. The analysis was based on Ensembl [32] (http://www.ensembl.org) genome annotations (versions: human 29, mouse 32, dog 34, and opossum 41). *d*_N_ and *d*_S_ statistics for retrocopy/parental gene pairs were estimated using the tool codeml as implemented in the PAML package [33] (http://abacus.gene.ucl.ac.uk/software/paml.html).

### Functional retrogenes.

For each species, we established a dataset enriched for functional retrogenes, i.e., retrocopies with intact ORFs and *d*_N_/*d*_S_ less than 0.5 (*p* < 0.05) in the comparison between the parental genes and retrogenes (suggesting purifying selection on both the parental and retrocopy sequence [1]). The test is based on a likelihood ratio test [34] that compares a codeml model in which *d*_N_/*d*_S_ is fixed to 0.5 (null model) to a model where *d*_N_/*d*_S_ is estimated from the data.

### Mapping retrocopies to Ensembl annotations.

Retrocopies were mapped to Ensembl annotations by overlapping the retrocopy coordinates with those from Ensembl exons.

### Linking expression data to parent–retrogene pairs.

We used normalized mouse microarray data generated in a previous study [13] for the parent–retrogene expression analyses. Parental genes were linked to probe sets using the Ensembl annotation provided by Affymetrix. Given that a number of retrocopies that integrated into introns of “host” genes are annotated in Ensembl as alternative splice variants of their host genes, we used a distinct procedure to link Affymetrix probe sets to retrocopies: First, we used BLAT ([35]) to map all probe sets onto the mouse genome sequence. A probe set was then assigned to a retrocopy if its best hit overlapped with the retrocopy. When multiple probe sets represented the expression of a retrocopy or a parental gene, we selected the probe set with the highest expression value in all testis measurements and considered it as representative. We excluded highly similar parent–retrogene pairs that may potentially cross-hybridize by requiring a minimum divergence at silent sites (*d*_S_) of 0.1. The overall procedure yielded expression data for 70 retrogenes and 116 parental genes (62 parent–retrogene pairs for which expression data are available for both members of the pair).

### Expression data analysis.

Expression data preprocessing, statistical filtering, gene clustering, and the testis-specificity determination procedure were established using the procedures described in [13]. In this study, probe sets were clustered and classified into four broad somatic (SO), mitotic (MI), meiotic (ME), and postmeiotic (PM) expression clusters, showing transcriptional peaks in Sertoli cells, spermatogonia, pachytene spermatocytes, and round spermatids, respectively. We empirically considered a gene to be significantly expressed, when its probe set had a signal greater than log2(100). We used two criteria to establish testis specificity for a gene. First, we chose all probe sets that are expressed in at least one male germline sample (Sertoli cells, spermatogonia, spermatocytes, spermatids, tubules, or total testis), but not in any somatic tissue analyzed (expression signals < log2(100)). Among these, we selected probe sets with expression signals that are at least 2-fold higher in the male germline sample(s) than in the somatic control samples.

### Phylogenetic dating of human retrocopies.

We dated human retrocopies by establishing the presence/absence of orthologous copies in the mouse, dog, opossum, and platypus genomes. For the therian genomes, we used a previously established procedure based on pairwise chained alignments of genomes (retrieved from the UCSC genome database, http://genome.ucsc.edu/) for this phylogenetic dating [2]. Briefly, we first extracted the best alignments that overlap with the genomic location of retrocopies and that are greater than 15 kb (this length ensures that the alignment also covers surrounding, non–retrocopy-derived sequences in the two species). We then scanned the alignments for aligned blocks that overlapped with the retrocopy. If the total length of the overlap corresponded to at least 60% of the length of the human retrocopy, the retrocopy was considered to be present in the other species. Conversely, when no such overlap was found, the retrocopy was assumed to be absent. Presence/absence of retrogenes shared between human and opossum in the platypus genome was established using a manual procedure, due to the incomplete assembly of this genome. Chained alignment data were visually inspected for the presence of significant blocks that overlap human retrocopies. Synteny of chains was validated by checking for the presence of genes in the flanks of the chains in platypus that are orthologous to the genes flanking the retrocopy in the human genome. Finally, the phylogenetic age of retrocopies was determined based on the pairwise presence/absence data obtained for all genomes; we assumed that a human retrocopy emerged on the branch before the divergence of the most-distant species in which its presence could be confirmed.

### Phylogenetic dating of lineage-specific retrocopies.

We used our automatic dating procedure (see above) to determine the presence/absence of mouse, dog, and opossum retrocopies in other therian genomes. Retrocopies with no orthologs detected were considered to be specific to the mouse, dog, or opossum lineage, respectively. In addition, we split the set of opossum-specific retrocopies into two subsets of retrocopies that are estimated to be generally older (parent–retrogene pairwise *d*_S_ > 0.5) or younger than 90 million years (parent–retrogene pairwise *d*_S_ < 0.5), which approximately corresponds to the human–dog lineage split time. The treshold *d*_S_ = 0.5 is assumed to roughly correspond to a divergence of 90 million years, as human–opossum orthologs have a median *d*_S_ ∼ 1, and the two species are estimated to have diverged about 180 Mya [36].

### Statistical tests.

We used standard Fisher exact and Mann-Whitney *U* tests. In addition, we used the resampling test described in [2] to assess the significance of the excess of parental genes on the X chromosome (the proportion of X-linked genes was set as the null expectation).

## Supporting Information

Figure S1 Independent Emergence of Functional Retrogenes from the Same Parents in the Eutherian and Marsupial Lineages Phylogenetic trees for *PGK* (A) or *CENTRIN* (B) parental genes and retrogenes are depicted. Protein sequences were aligned, and the resulting alignments were used to guide coding sequence alignments. The most likely trees based on the coding sequence alignments were inferred using MrBayes and plotted with the FigTree software. Gene IDs refer to Tables S1 (Homo sapiens genes), S2 (Mus musculus genes), S3 (Canis familiaris genes), or S4 (Monodelphis domestica genes).
(109 KB PDF)

Table S1 Human Retrogene Set (250 KB DOC)

Table S2 Mouse Retrogene Set (366 KB DOC)

Table S3 Dog Retrogene Set (259 KB DOC)

Table S4 Opossum Retrogene Set (369 KB DOC)

Table S5 Mouse Parental Gene and Retrogene Expression Data (395 KB DOC)

## Abbreviations

MSCI: male meiotic sex chromosome inactivation
Mya: million years ago
XCI: X chromosome inactivation
XCR: X conserved region
