# Supplementary material for: Chromosomal Gene Movements Reflect the Recent Origin and Biology of Therian Sex Chromosomes
Source: PLoS Biol. 2008 Apr 1;6(4):e80. doi: 10.1371/journal.pbio.0060080 (PMC2276528; doi:10.1371/journal.pbio.0060080)
Supplement: Supplementary file 1 [file pbio.0060080.sg001.pdf]

A

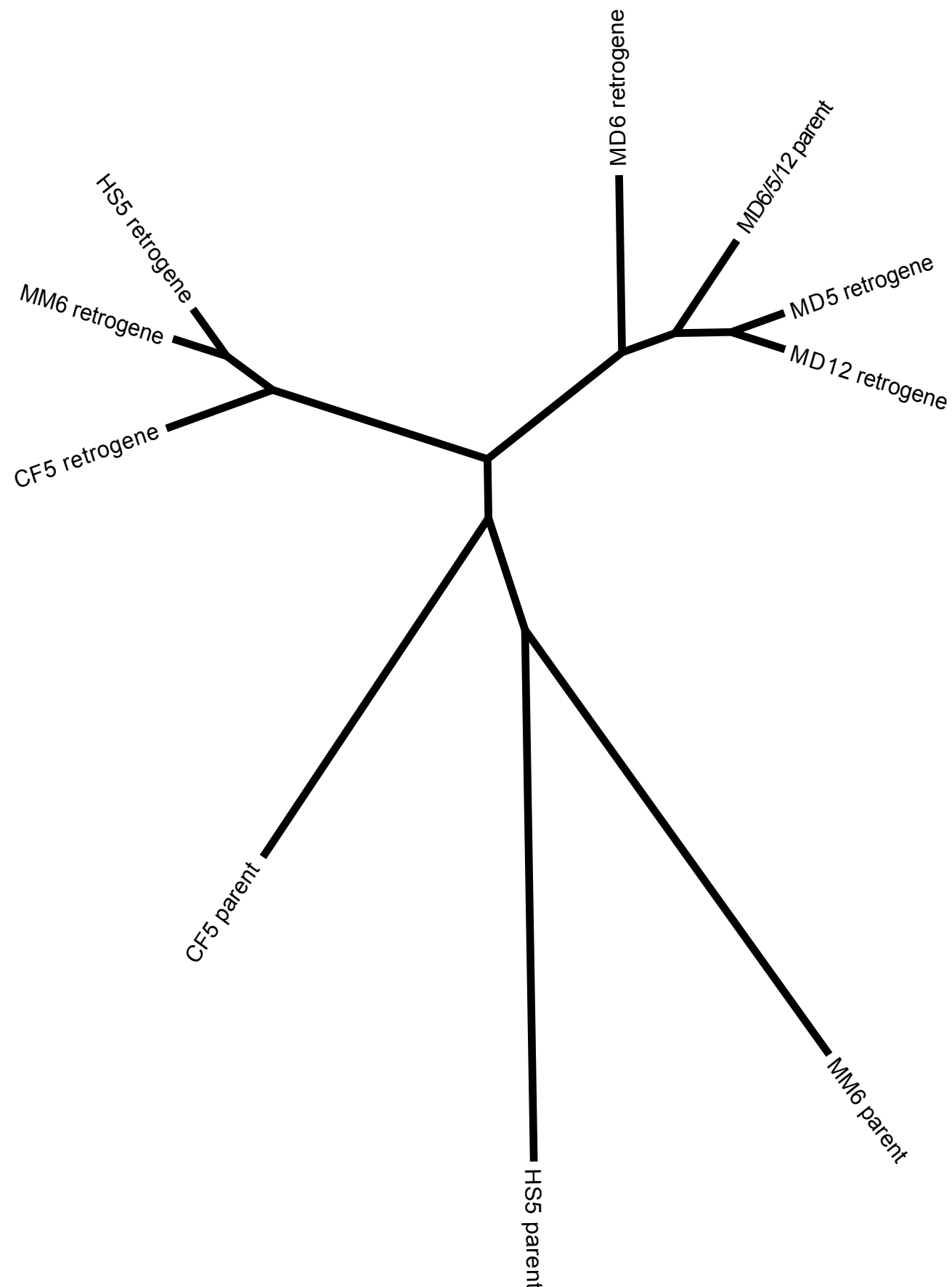
MD6 retrogene
MD6/5/12 parent
MD5 retrogene
MD12 retrogene
HS5 retrogene
MM6 retrogene
CF5 retrogene
CF5 parent
HS5 parent
MM6 parent

B

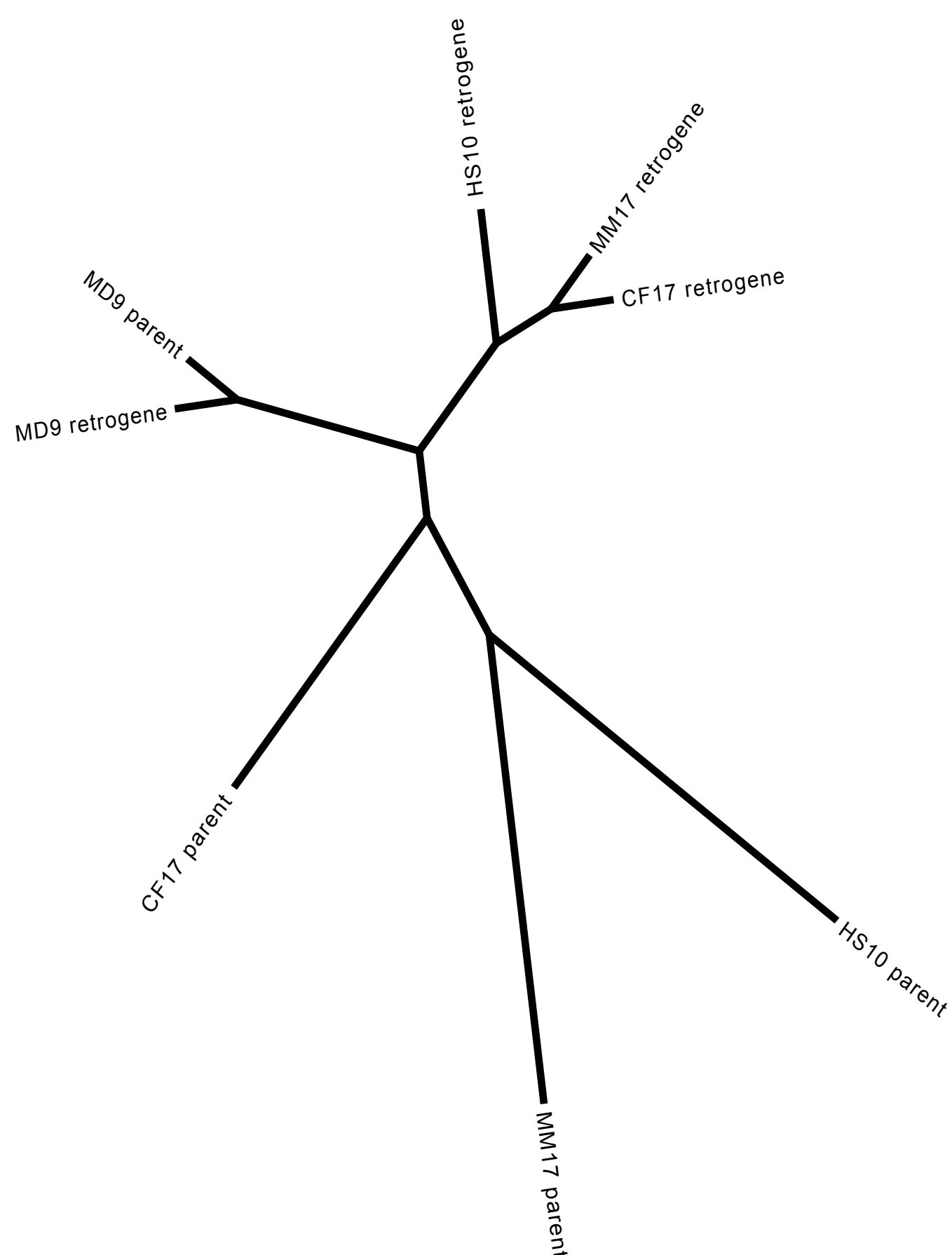
HS10 retrogene
MM17 retrogene
CF17 retrogene
MD9 parent
MD9 retrogene
CF17 parent
MM17 parent
HS10 parent
