# Supplementary material for: Chromosomal Gene Movements Reflect the Recent Origin and Biology of Therian Sex Chromosomes
Source: PLoS Biol. 2008 Apr 1;6(4):e80. doi: 10.1371/journal.pbio.0060080 (PMC2276528; doi:10.1371/journal.pbio.0060080)
Supplement: Supplementary file 2 [file pbio.0060080.st001.doc]

**Table S1.** Human retrogene set.

| Retro. ID | Retro. name^1^ | Retro. ENSG^2^ | Retro. ENSP^2^ | Retro. chr. | Retro. strand | Retro. start | Retro. end | Par. name^1^ | Par. ENSG^2^ | Par. ENSP^2^ | Par. chr. | *d*_N_/*d*_S_ | *d*_S_ | Age^3^ |
| --- | --- | --- | --- | --- | --- | --- | --- | --- | --- | --- | --- | --- | --- | --- |
| HS1 | *KLHL9* | ENSG00000198642 | ENSP00000351933 | 9 | - | 21323021 | 21324832 | *KLHL13* | ENSG00000003096 | ENSP00000262820 | X | 0.05 | 0.65 | *C* |
| HS2 | *RRAGA* | ENSG00000155876 | ENSP00000286334 | 9 | + | 19039656 | 19040597 | *RRAGB* | ENSG00000083750 | ENSP00000262851 | X | 0.01 | 1.45 | *C* |
| HS3 | *NXT1* | ENSG00000132661 | ENSP00000254998 | 20 | + | 23282680 | 23283099 | *NXT2* | ENSG00000101888 | ENSP00000218004 | X | 0.11 | 1.37 | *C* |
| HS4 | *ARD1B* | ENSG00000156269 | ENSP00000286794 | 4 | - | 80603525 | 80604211 | *ARD1* | ENSG00000102030 | ENSP00000218149 | X | 0.09 | 0.97 | *C* |
| HS5 | *PGK2* | ENSG00000170950 | ENSP00000305995 | 6 | - | 49861610 | 49862860 | *PGK1* | ENSG00000102144 | ENSP00000218265 | X | 0.11 | 0.58 | *C* |
| HS6 | *TAF7* | ENSG00000178913 | ENSP00000312709 | 5 | - | 140678804 | 140679796 | *TAF7L* | ENSG00000102387 | ENSP00000349235 | X | 0.22 | 1.30 | *C* |
| HS7 | *RPL36AL* | ENSG00000165502 | ENSP00000346012 | 14 | - | 49155256 | 49155573 | *RPL36A* | ENSG00000102391 | ENSP00000346013 | X | 0.01 | 0.70 | *C* |
| HS8 | *PDHA2* | ENSG00000163114 | ENSP00000295266 | 4 | + | 97118472 | 97119644 | *PDHA1* | ENSG00000131828 | ENSP00000254142 | X | 0.14 | 0.58 | *C* |
| HS9 | *PRPS1L1* | ENSG00000153287 | ENSP00000283289 | 7 | - | 17839693 | 17840643 | *PRPS1* | ENSG00000147224 | ENSP00000276174 | X | 0.11 | 0.25 | *C* |
| HS10 | *CETN1* | ENSG00000177143 | ENSP00000319052 | 18 | + | 570410 | 570925 | *CETN2* | ENSG00000147400 | ENSP00000276365 | X | 0.02 | 4.41 | *C* |
| HS11 | *RPL10L* | ENSG00000165496 | ENSP00000298283 | 14 | - | 46190049 | 46190690 | *RPL10* | ENSG00000147403 | ENSP00000341730 | X | 0.04 | 0.55 | *C* |
| HS12 | *FAM11B* | ENSG00000179724 | ENSP00000314309 | 2 | - | 120695734 | 120696783 | *FAM11A* | ENSG00000155984 | ENSP00000286456 | X | 0.03 | 1.79 | *C* |
| HS13 | *TAF9* | ENSG00000085231 | ENSP00000217893 | 5 | - | 68696557 | 68697321 | *TAF9L* | ENSG00000187325 | ENSP00000337534 | X | 0.13 | 0.71 | *C* |
| HS14 | *NUP62* | ENSG00000104951 | ENSP00000305503 | 19 | - | 55103312 | 55104739 | *NUP62CL* | ENSG00000198088 | ENSP00000348914 | X | 0.00 | 63.19 | *C* |
| HS15 | *RBMXL2* | ENSG00000170748 | ENSP00000304139 | 11 | + | 7066929 | 7068086 | *RBMX* | ENSG00000147274 | ENSP00000276230 | X | 0.05 | 3.20 | *D* |
| HS16 | *-* | - | - | 20 | + | 29598963 | 29599511 | *MCTS1* | ENSG00000101898 | ENSP00000218014 | X | 0.09 | 0.26 | *D* |
| HS17 | *FAM50B* | ENSG00000145945 | ENSP00000274639 | 6 | + | 3795046 | 3796020 | *FAM50A* | ENSG00000071859 | ENSP00000158526 | X | 0.00 | 23.70 | *D* |
| HS18 | *-* | - | - | 14 | + | 64805301 | 64805570 | *RPL36A* | ENSG00000102391 | ENSP00000346013 | X | 0.06 | 1.86 | *E* |
| HS19 | *-* | ENSG00000175090 | ENSP00000352977 | 17 | - | 56159312 | 56159626 | *RPL36A* | ENSG00000102391 | ENSP00000346013 | X | 0.07 | 0.81 | *E* |
| HS20 |  | ENSG00000152433 | ENSP00000310153 | 19 | + | 62568015 | 62568434 | *TRAPPC2* | ENSG00000196459 | ENSP00000350966 | X | 0.09 | 0.08 | *E* |
| HS21 | *GKP3* | ENSG00000170095 | ENSP00000302036 | 4 | - | 166556745 | 166558403 | *GK* | ENSG00000198814 | ENSP00000354642 | X | 0.06 | 0.07 | *E* |
| HS22 | *-* | ENSG00000148944 | ENSP00000351630 | 11 | - | 25566601 | 25566918 | *RPL36A* | ENSG00000102391 | ENSP00000346013 | X | 0.05 | 1.12 | *E* |
| HS23 | *-* | ENSG00000180574 | ENSP00000323063 | 12 | + | 10549770 | 10551185 | *EIF2S3* | ENSG00000130741 | ENSP00000253039 | X | 0.15 | 0.05 | *E* |
| HS24 | *-* | ENSG00000182447 | ENSP00000330808 | 3 | + | 162703510 | 162704403 | *-* | ENSG00000182957 | ENSP00000333737 | 13 | 0.01 | 66.07 | *A* |
| HS25 | *-* | - | - | 6 | + | 31903708 | 31905630 | *HSPA8* | ENSG00000109971 | ENSP00000227378 | 11 | 0.00 | 24.69 | *A* |
| HS26 | *-* | - | - | 6 | - | 31885807 | 31887729 | *HSPA8* | ENSG00000109971 | ENSP00000227378 | 11 | 0.00 | 30.49 | *A* |
| HS27 | *-* | ENSG00000165527 | ENSP00000298316 | 14 | + | 49430185 | 49430730 | *-* | ENSG00000134287 | ENSP00000256682 | 12 | 0.00 | 49.76 | *A* |
| HS28 | *HSPA2* | ENSG00000126803 | ENSP00000247207 | 14 | + | 64077322 | 64079238 | *HSPA8* | ENSG00000109971 | ENSP00000227378 | 11 | 0.00 | 16.52 | *A* |
| HS29 | *DNAJB8* | ENSG00000179407 | ENSP00000316053 | 3 | - | 129664092 | 129664787 | *DNAJB6* | ENSG00000105993 | ENSP00000262177 | 7 | 0.00 | 65.14 | *A* |
| HS30 | *-* | ENSG00000184860 | ENSP00000332407 | 16 | - | 80590251 | 80591336 | *-* | ENSG00000183921 | ENSP00000330812 | 16 | 0.02 | 23.13 | *A* |
| HS31 | *-* | - | - | 5 | - | 179038311 | 179038658 | *PGEA1* | ENSG00000100211 | ENSP00000216029 | 22 | 0.01 | 78.80 | *A* |
| HS32 | *-* | ENSG00000182431 | ENSP00000335773 | 5 | - | 17544471 | 17544878 | *-* | ENSG00000163041 | ENSP00000295172 | 1 | 0.07 | 1.41 | *A* |
| HS33 | *-* | - | - | 21 | + | 34389372 | 34391519 | *SLC5A1* | ENSG00000100170 | ENSP00000266088 | 22 | 0.01 | 62.85 | *A* |
| HS34 | *SOD3* | ENSG00000109610 | ENSP00000226877 | 4 | + | 24477462 | 24478133 | *SOD1* | ENSG00000142168 | ENSP00000270142 | 21 | 0.01 | 67.97 | *A* |
| HS35 | *C20orf135* | ENSG00000183260 | ENSP00000329892 | 20 | + | 61963339 | 61964745 | *BAT5* | ENSG00000096467 | ENSP00000293664 | 6 | 0.03 | 9.40 | *A* |
| HS36 | *-* | - | - | 12 | - | 88419044 | 88420615 | *GALNT12* | ENSG00000119514 | ENSP00000238467 | 9 | 0.01 | 29.33 | *A* |
| HS37 | *CALML3* | ENSG00000178363 | ENSP00000315299 | 10 | + | 5557008 | 5557496 | *-* | ENSG00000160014 | ENSP00000291295 | 19 | 0.01 | 8.75 | *A* |
| HS38 | *SFN* | ENSG00000175793 | ENSP00000340989 | 1 | + | 26873847 | 26874581 | *-* | ENSG00000164924 | ENSP00000309503 | 8 | 0.01 | 14.44 | *A* |
| HS39 | *CALML5* | ENSG00000178372 | ENSP00000315372 | 10 | - | 5530965 | 5531426 | *-* | ENSG00000160014 | ENSP00000291295 | 19 | 0.02 | 21.76 | *A* |
| HS40 | *-* | - | - | 6 | + | 31891496 | 31893436 | *HSPA8* | ENSG00000109971 | ENSP00000227378 | 11 | 0.00 | 25.04 | *A* |
| HS41 | *-* | ENSG00000184378 | ENSP00000333037 | 3 | - | 170967926 | 170968843 | *-* | ENSG00000075624 | ENSP00000158302 | 7 | 0.02 | 17.68 | *B* |
| HS42 | *HTR1A* | ENSG00000178394 | ENSP00000316244 | 5 | - | 63292038 | 63293303 | *-* | ENSG00000149295 | ENSP00000278597 | 11 | 0.01 | 98.25 | *B* |
| HS43 | *SLC35A4* | ENSG00000176087 | ENSP00000327133 | 5 | + | 139926940 | 139927911 | *SLC35A3* | ENSG00000117620 | ENSP00000326111 | 1 | 0.02 | 53.47 | *B* |
| HS44 | *TACSTD2* | ENSG00000184292 | ENSP00000327831 | 1 | - | 58753882 | 58754778 | *TACSTD1* | ENSG00000119888 | ENSP00000263735 | 2 | 0.01 | 69.99 | *B* |
| HS45 | *RHOH* | ENSG00000168421 | ENSP00000303689 | 4 | + | 40067556 | 40068146 | *-* | ENSG00000136238 | ENSP00000258737 | 7 | 0.03 | 16.13 | *B* |
| HS46 | *-* | ENSG00000177105 | ENSP00000339467 | 11 | - | 3805373 | 3805981 | *-* | ENSG00000136238 | ENSP00000258737 | 7 | 0.00 | 66.68 | *B* |
| HS47 | *ALDH1B1* | ENSG00000137124 | ENSP00000259658 | 9 | + | 38385747 | 38387297 | *ALDH2* | ENSG00000111275 | ENSP00000261733 | 12 | 0.06 | 3.02 | *B* |
| HS48 | *-* | ENSG00000182107 | ENSP00000347930 | 14 | - | 60816579 | 60817586 | *TMEM30A* | ENSG00000112697 | ENSP00000323733 | 6 | 0.01 | 65.42 | *B* |
| HS49 | *-* | ENSG00000169067 | ENSP00000306469 | 5 | - | 56813165 | 56814292 | *-* | ENSG00000075624 | ENSP00000158302 | 7 | 0.02 | 2.73 | *B* |
| HS50 | *HSPA7* | ENSG00000173110 | ENSP00000310219 | 1 | + | 158307502 | 158309433 | *HSPA8* | ENSG00000109971 | ENSP00000227378 | 11 | 0.01 | 21.86 | *B* |
| HS51 | *-* | ENSG00000196289 | ENSP00000348342 | 1 | + | 238447120 | 238449024 | *BECN1* | ENSG00000126581 | ENSP00000246933 | 17 | 0.26 | 1.24 | *C* |
| HS52 | *-* | ENSG00000186222 | ENSP00000318128 | 4 | + | 6836010 | 6836660 | *-* | ENSG00000124243 | ENSP00000341676 | 20 | 0.07 | 6.31 | *C* |
| HS53 | *-* | ENSG00000172404 | ENSP00000307197 | 22 | - | 39581573 | 39582499 | *DNAJB6* | ENSG00000105993 | ENSP00000262177 | 7 | 0.32 | 0.91 | *C* |
| HS54 | *-* | ENSG00000143878 | ENSP00000272233 | 2 | + | 20568838 | 20569443 | *-* | ENSG00000067560 | ENSP00000265538 | 3 | 0.00 | 57.36 | *C* |
| HS55 | *WDR5B* | ENSG00000196981 | ENSP00000330381 | 3 | - | 123616080 | 123616982 | *-* | ENSG00000196363 | ENSP00000351446 | 9 | 0.05 | 1.04 | *C* |
| HS56 | *PCBP1* | ENSG00000169564 | ENSP00000305556 | 2 | + | 70226528 | 70227595 | *PCBP2* | ENSG00000197111 | ENSP00000352228 | 12 | 0.11 | 0.72 | *C* |
| HS57 | *PPP3R2* | ENSG00000188386 | ENSP00000342600 | 9 | - | 101436250 | 101436768 | *-* | ENSG00000115953 | ENSP00000234310 | 2 | 0.03 | 3.05 | *C* |
| HS58 | *-* | ENSG00000170967 | ENSP00000302805 | 11 | + | 103412762 | 103413949 | *-* | ENSG00000198031 | ENSP00000317681 | 1 | 0.11 | 1.50 | *C* |
| HS59 | *HMGN4* | ENSG00000182952 | ENSP00000327691 | 6 | + | 26653415 | 26653684 | *HMGN2* | ENSG00000198830 | ENSP00000355228 | 1 | 0.17 | 0.46 | *C* |
| HS60 | *-* | ENSG00000188573 | ENSP00000339522 | 5 | + | 167889377 | 167890066 | *FBL* | ENSG00000105202 | ENSP00000221801 | 19 | 0.02 | 4.22 | *C* |
| HS61 | *-* | ENSG00000170777 | ENSP00000341677 | 9 | + | 6318537 | 6319016 | *-* | ENSG00000101150 | ENSP00000351350 | 20 | 0.28 | 0.91 | *C* |
| HS62 | *MKRN3* | ENSG00000179455 | ENSP00000313881 | 15 | + | 21362102 | 21363544 | *MKRN1* | ENSG00000133606 | ENSP00000255977 | 7 | 0.36 | 1.00 | *C* |
| HS63 | *HNRPF* | ENSG00000169813 | ENSP00000338477 | 10 | - | 43202095 | 43203339 | *HNRPH1* | ENSG00000169045 | ENSP00000349573 | 5 | 0.12 | 1.11 | *C* |
| HS64 | *FTMT* | ENSG00000181867 | ENSP00000313691 | 5 | + | 121215559 | 121216284 | *FTH1* | ENSG00000167996 | ENSP00000301775 | 11 | 0.07 | 1.76 | *C* |
| HS65 | *ATP6V1E2* | ENSG00000171142 | ENSP00000304891 | 2 | - | 46650825 | 46651502 | *ATP6V1E1* | ENSG00000131100 | ENSP00000253413 | 22 | 0.11 | 1.30 | *C* |
| HS66 | *SLC25A2* | ENSG00000120329 | ENSP00000239451 | 5 | - | 140662715 | 140663617 | *SLC25A15* | ENSG00000102743 | ENSP00000342267 | 13 | 0.18 | 0.37 | *C* |
| HS67 | *RANBP6* | ENSG00000137040 | ENSP00000259569 | 9 | - | 6002294 | 6005608 | *RANBP5* | ENSG00000065150 | ENSP00000350219 | 13 | 0.17 | 0.70 | *C* |
| HS68 | *-* | ENSG00000151005 | ENSP00000280605 | 4 | - | 164750615 | 164752492 | *TKT* | ENSG00000163931 | ENSP00000296289 | 3 | 0.03 | 8.06 | *D* |
| HS69 | *OXCT2* | ENSG00000198754 | ENSP00000355412 | 1 | - | 39904474 | 39906021 | *OXCT1* | ENSG00000083720 | ENSP00000196371 | 5 | 0.05 | 2.91 | *D* |
| HS70 | *CSNK1A1L* | ENSG00000180138 | ENSP00000322723 | 13 | - | 36576384 | 36577418 | *-* | ENSG00000113712 | ENSP00000261798 | 5 | 0.20 | 0.23 | *E* |
| HS71 | *-* | ENSG00000136149 | ENSP00000351449 | 13 | - | 53912844 | 53913452 | *RPL13A* | ENSG00000142541 | ENSP00000270634 | 19 | 0.16 | 0.07 | *E* |
| HS72 | *-* | - | - | 2 | + | 242137821 | 242138396 | *KCTD5* | ENSG00000167977 | ENSP00000301738 | 16 | 0.25 | 0.37 | *E* |
| HS73 | *-* | ENSG00000197149 | ENSP00000351543 | 12 | + | 117146610 | 117147488 | *RPS2* | ENSG00000140988 | ENSP00000346031 | 16 | 0.12 | 0.04 | *E* |
| HS74 | *-* | ENSG00000173180 | ENSP00000351523 | 6 | + | 114267178 | 114267498 | *-* | ENSG00000156482 | ENSP00000287038 | 8 | 0.13 | 0.34 | *E* |
| HS75 | *-* | ENSG00000188181 | ENSP00000343513 | 19 | + | 14042211 | 14043050 | *EEF1D* | ENSG00000104529 | ENSP00000220971 | 8 | 0.18 | 0.12 | *E* |
| HS76 | *PRKACG* | ENSG00000165059 | ENSP00000297734 | 9 | - | 68857511 | 68858563 | *PRKACA* | ENSG00000072062 | ENSP00000309591 | 19 | 0.12 | 0.76 | *E* |
| HS77 | *-* | ENSG00000188450 | ENSP00000345158 | 11 | + | 18167124 | 18167747 | *-* | ENSG00000139433 | ENSP00000315263 | 12 | 0.22 | 0.12 | *E* |
| HS78 | *-* | ENSG00000182382 | ENSP00000349308 | 11 | + | 17206511 | 17207272 | *RPS2* | ENSG00000140988 | ENSP00000341885 | 16 | 0.25 | 0.12 | *E* |
| HS79 | *-* | ENSG00000187072 | ENSP00000333957 | 11 | - | 129407973 | 129408326 | *TCEB2* | ENSG00000103363 | ENSP00000262306 | 16 | 0.10 | 0.27 | *E* |
| HS80 | *-* | ENSG00000180153 | ENSP00000322781 | 10 | + | 23465812 | 23466594 | *-* | ENSG00000164924 | ENSP00000309503 | 8 | 0.16 | 0.07 | *E* |
| HS81 | *-* | ENSG00000196183 | ENSP00000353405 | 14 | - | 104373380 | 104374267 | *RPS2* | ENSG00000140988 | ENSP00000346031 | 16 | 0.21 | 0.12 | *E* |
| HS82 | *LDHAL6B* | ENSG00000171989 | ENSP00000302393 | 15 | + | 57286433 | 57287575 | *LDHAL6A* | ENSG00000166800 | ENSP00000280706 | 11 | 0.31 | 0.32 | *E* |
| HS83 | *-* | ENSG00000184637 | ENSP00000332264 | 17 | - | 4706435 | 4706866 | *ATP6V0C* | ENSG00000185883 | ENSP00000329757 | 16 | 0.26 | 0.54 | *E* |
| HS84 | *-* | ENSG00000169253 | ENSP00000347064 | 4 | - | 77315086 | 77315397 | *RPL36* | ENSG00000130255 | ENSP00000252543 | 19 | 0.13 | 0.67 | *E* |
| HS85 | *-* | ENSG00000164159 | ENSP00000350499 | 4 | - | 144628037 | 144628831 | *RPS2* | ENSG00000140988 | ENSP00000346031 | 16 | 0.14 | 0.08 | *E* |
| HS86 | *-* | ENSG00000185956 | ENSP00000329099 | 22 | + | 23432814 | 23433041 | *CRIP1* | ENSG00000182351 | ENSP00000332449 | 14 | 0.14 | 0.21 | *E* |
| HS87 | *-* | ENSG00000198618 | ENSP00000351238 | 21 | + | 19151966 | 19152463 | *-* | ENSG00000196262 | ENSP00000348240 | 7 | 0.00 | 0.02 | *E* |
| HS88 | *-* | - | - | 20 | + | 48670704 | 48670976 | *RPL36* | ENSG00000130255 | ENSP00000252543 | 19 | 0.15 | 0.50 | *E* |
| HS89 | *-* | ENSG00000177835 | ENSP00000325352 | 2 | + | 210499035 | 210499808 | *SNAI1* | ENSG00000124216 | ENSP00000244050 | 20 | 0.28 | 0.32 | *E* |
| HS90 | *-* | ENSG00000144332 | ENSP00000272762 | 2 | + | 180504485 | 180504883 | *-* | ENSG00000141759 | ENSP00000269601 | 18 | 0.23 | 0.44 | *E* |
| HS91 | *-* | - | - | 2 | + | 85054702 | 85055445 | *RPS2* | ENSG00000140988 | ENSP00000346031 | 16 | 0.04 | 0.12 | *E* |
| HS92 | *-* | ENSG00000170793 | ENSP00000348088 | 2 | - | 47829510 | 47830040 | *RPL18A* | ENSG00000105640 | ENSP00000222247 | 19 | 0.12 | 0.10 | *E* |
| HS93 | *-* | ENSG00000176093 | ENSP00000352313 | 18 | - | 35168838 | 35169599 | *-* | ENSG00000148303 | ENSP00000340366 | 9 | 0.18 | 0.07 | *E* |
| HS94 | *-* | ENSG00000186080 | ENSP00000334513 | 9 | + | 99146950 | 99148446 | *KRT8* | ENSG00000170421 | ENSP00000293308 | 12 | 0.21 | 0.25 | *E* |

^1^ Names for retrogenes and parental genes are from HUGO.

^2^ ENSG and ENSP indicate Ensembl identifiers for genes and proteins, respectively.

^3^ Ages of retrogenes are indicated according to branch labels defined in Figure 1.
