# Supplementary material for: Chromosomal Gene Movements Reflect the Recent Origin and Biology of Therian Sex Chromosomes
Source: PLoS Biol. 2008 Apr 1;6(4):e80. doi: 10.1371/journal.pbio.0060080 (PMC2276528; doi:10.1371/journal.pbio.0060080)
Supplement: Supplementary file 3 [file pbio.0060080.st002.doc]

**Table S2.** Mouse retrogene set.

| Retro. ID | Retro. ENSG^1^ | Retro. ENSP^1^ | Retro. chr. | Retro. strand | Retro. start | Retro. end | Par. ENSG^1^ | Par. ENSP^1^ | Par. chr. | *d*_N_/*d*_S_ | *d*_S_ | Human shared^2^ |
| --- | --- | --- | --- | --- | --- | --- | --- | --- | --- | --- | --- | --- |
| MM1 | ENSMUSG00000039224 | ENSMUSP00000035261 | 1 | + | 186475492 | 186477471 | ENSMUSG00000000787 | ENSMUSP00000000804 | X | 0.04 | 0.62 | - |
| MM2 | ENSMUSG00000046292 | ENSMUSP00000059358 | 12 | + | 31599912 | 31600865 | ENSMUSG00000031432 | ENSMUSP00000033809 | X | 0.04 | 0.69 | + |
| MM3 | ENSMUSG00000023350 | ENSMUSP00000024121 | 17 | - | 30888656 | 30891379 | ENSMUSG00000041229 | ENSMUSP00000040765 | X | 0.35 | 0.60 | und |
| MM4 | ENSMUSG00000036992 | ENSMUSP00000038329 | 2 | + | 148132278 | 148132697 | ENSMUSG00000042271 | ENSMUSP00000048221 | X | 0.09 | 1.80 | + |
| MM5 | - | - | 2 | + | 152144208 | 152144750 | ENSMUSG00000000355 | ENSMUSP00000000365 | X | 0.03 | 0.92 | + |
| MM6 | ENSMUSG00000031233 | ENSMUSP00000033585 | 3 | - | 7836561 | 7837811 | ENSMUSG00000062070 | ENSMUSP00000080302 | X | 0.09 | 1.12 | - |
| MM7 | ENSMUSG00000055891 | - | 3 | - | 107350365 | 107350790 | ENSMUSG00000015290 | ENSMUSP00000015434 | X | 0.01 | 58.10 | + |
| MM8 | ENSMUSG00000047674 | ENSMUSP00000060774 | 3 | - | 140175526 | 140176698 | ENSMUSG00000031299 | ENSMUSP00000033662 | X | 0.10 | 1.39 | + |
| MM9 | ENSMUSG00000050553 | ENSMUSP00000052226 | 5 | - | 96473413 | 96475074 | ENSMUSG00000025059 | ENSMUSP00000076183 | X | 0.12 | 0.81 | + |
| MM10 | ENSMUSG00000046717 | ENSMUSP00000050818 | 6 | - | 139449375 | 139450403 | ENSMUSG00000031221 | ENSMUSP00000033570 | X | 0.19 | 1.15 | - |
| MM11 | ENSMUSG00000051956 | ENSMUSP00000066906 | 6 | - | 23695652 | 23696797 | ENSMUSG00000031438 | ENSMUSP00000033815 | X | 0.15 | 2.17 | + |
| MM12 | ENSMUSG00000063171 | ENSMUSP00000071658 | 6 | + | 149288533 | 149289318 | ENSMUSG00000031320 | ENSMUSP00000033683 | X | 0.01 | 3.31 | - |
| MM13 | - | - | 7 | + | 101158652 | 101159740 | ENSMUSG00000031134 | ENSMUSP00000033470 | X | 0.01 | 10.63 | + |
| MM14 | - | - | 7 | + | 44757316 | 44758392 | ENSMUSG00000002010 | ENSMUSP00000056502 | X | 0.16 | 1.31 | + |
| MM15 | ENSMUSG00000044068 | ENSMUSP00000062025 | 11 | + | 22868019 | 22869302 | ENSMUSG00000031370 | ENSMUSP00000033736 | X | 0.18 | 0.88 | - |
| MM16 | ENSMUSG00000038246 | ENSMUSP00000046980 | 13 | + | 34278720 | 34279721 | ENSMUSG00000001962 | ENSMUSP00000002027 | X | 0.01 | 8.65 | + |
| MM17 | ENSMUSG00000050996 | ENSMUSP00000057392 | 18 | - | 9664247 | 9664762 | ENSMUSG00000031347 | ENSMUSP00000033713 | X | 0.05 | 2.17 | + |
| MM18 | ENSMUSG00000032883 | ENSMUSP00000045291 | 1 | + | 78998207 | 79000489 | ENSMUSG00000063785 | ENSMUSP00000079538 | X | 0.33 | 0.64 | und |
| MM19 | - | - | 10 | - | 108585735 | 108586493 | ENSMUSG00000031201 | ENSMUSP00000033544 | X | 0.21 | 0.53 | - |
| MM20 | ENSMUSG00000044524 | ENSMUSP00000055882 | 12 | + | 96591073 | 96594738 | ENSMUSG00000034311 | ENSMUSP00000048383 | X | 0.24 | 0.64 | - |
| MM21 | ENSMUSG00000057762 | ENSMUSP00000071119 | 13 | - | 93288071 | 93288526 | ENSMUSG00000031146 | ENSMUSP00000033486 | X | 0.00 | 0.03 | - |
| MM22 | ENSMUSG00000056584 | ENSMUSP00000068992 | 3 | - | 40329322 | 40329873 | ENSMUSG00000031198 | ENSMUSP00000033541 | X | 0.10 | 0.79 | - |
| MM23 | ENSMUSG00000059335 | - | 4 | - | 87706357 | 87708195 | ENSMUSG00000036782 | ENSMUSP00000041190 | X | 0.04 | 1.20 | + |
| MM24 | ENSMUSG00000048363 | ENSMUSP00000053392 | 5 | + | 103553870 | 103558501 | ENSMUSG00000037475 | ENSMUSP00000044677 | X | 0.12 | 0.61 | und |
| MM25 | ENSMUSG00000048591 | ENSMUSP00000062195 | 1 | - | 88024059 | 88024784 | ENSMUSG00000029131 | ENSMUSP00000008733 | 5 | 0.03 | 6.33 | + |
| MM26 | ENSMUSG00000041483 | ENSMUSP00000039003 | 1 | + | 136474173 | 136476743 | ENSMUSG00000022811 | ENSMUSP00000087106 | 16 | 0.07 | 7.70 | + |
| MM27 | ENSMUSG00000046934 | ENSMUSP00000052373 | 10 | - | 99737819 | 99739216 | ENSMUSG00000005683 | ENSMUSP00000005826 | 10 | 0.21 | 0.19 | - |
| MM28 | - | - | 10 | + | 82753948 | 82754865 | ENSMUSG00000040331 | ENSMUSP00000073341 | 7 | 0.25 | 1.56 | + |
| MM29 | ENSMUSG00000045048 | ENSMUSP00000050015 | 10 | + | 56909190 | 56909888 | ENSMUSG00000046027 | ENSMUSP00000074872 | 7 | 0.03 | 22.61 | - |
| MM30 | - | - | 11 | - | 81298321 | 81299133 | ENSMUSG00000038845 | ENSMUSP00000047536 | 11 | 0.14 | 0.24 | - |
| MM31 | ENSMUSG00000061519 | ENSMUSP00000074001 | 11 | - | 96616530 | 96616970 | ENSMUSG00000063789 | ENSMUSP00000080608 | 8 | 0.08 | 0.24 | - |
| MM32 | ENSMUSG00000063129 | ENSMUSP00000079022 | 12 | + | 52285674 | 52286765 | ENSMUSG00000030695 | ENSMUSP00000032934 | 7 | 0.09 | 0.30 | - |
| MM33 | ENSMUSG00000044147 | ENSMUSP00000055862 | 12 | + | 66204571 | 66205116 | ENSMUSG00000051853 | ENSMUSP00000050689 | 15 | 0.01 | 38.22 | + |
| MM34 | ENSMUSG00000044444 | ENSMUSP00000054053 | 13 | - | 54024251 | 54024661 | ENSMUSG00000018293 | ENSMUSP00000018437 | 11 | 0.01 | 64.24 | + |
| MM35 | ENSMUSG00000044250 | ENSMUSP00000055485 | 15 | + | 97460726 | 97462024 | ENSMUSG00000037773 | ENSMUSP00000087009 | 2 | 0.03 | 12.46 | + |
| MM36 | ENSMUSG00000034379 | ENSMUSP00000040852 | 16 | + | 34822921 | 34823904 | ENSMUSG00000026917 | ENSMUSP00000028281 | 2 | 0.08 | 1.32 | + |
| MM37 | ENSMUSG00000053375 | ENSMUSP00000065285 | 17 | - | 84800980 | 84801669 | ENSMUSG00000019210 | ENSMUSP00000019354 | 6 | 0.06 | 3.50 | + |
| MM38 | - | - | 17 | - | 5324892 | 5325887 | ENSMUSG00000030851 | ENSMUSP00000014545 | 7 | 0.17 | 1.68 | + |
| MM39 | ENSMUSG00000044526 | ENSMUSP00000059715 | 17 | - | 54148366 | 54149346 | ENSMUSG00000036503 | ENSMUSP00000049331 | 3 | 0.01 | 63.33 | - |
| MM40 | ENSMUSG00000050612 | ENSMUSP00000054909 | 17 | - | 63346975 | 63348477 | ENSMUSG00000028367 | ENSMUSP00000030051 | 4 | 0.06 | 5.47 | + |
| MM41 | ENSMUSG00000046196 | ENSMUSP00000053781 | 17 | + | 78033483 | 78035120 | ENSMUSG00000038172 | ENSMUSP00000040590 | 4 | 0.20 | 2.51 | + |
| MM42 | ENSMUSG00000067416 | - | 17 | + | 31125053 | 31126150 | ENSMUSG00000062825 | ENSMUSP00000071486 | 11 | 0.01 | 69.76 | + |
| MM43 | ENSMUSG00000050304 | ENSMUSP00000052849 | 18 | - | 37861403 | 37862296 | ENSMUSG00000031482 | ENSMUSP00000033871 | 8 | 0.15 | 1.05 | + |
| MM44 | ENSMUSG00000051732 | ENSMUSP00000066639 | 18 | + | 39997483 | 39999366 | ENSMUSG00000022283 | ENSMUSP00000001809 | 15 | 0.17 | 0.62 | - |
| MM45 | ENSMUSG00000024510 | ENSMUSP00000025388 | 18 | + | 52546949 | 52547731 | ENSMUSG00000024661 | ENSMUSP00000025563 | 19 | 0.02 | 5.02 | + |
| MM46 | ENSMUSG00000058667 | ENSMUSP00000044900 | 19 | - | 29062236 | 29065550 | ENSMUSG00000030662 | ENSMUSP00000085742 | 14 | 0.12 | 1.12 | + |
| MM47 | ENSMUSG00000050370 | ENSMUSP00000049683 | 19 | - | 33805413 | 33806306 | ENSMUSG00000031604 | ENSMUSP00000034015 | 8 | 0.01 | 64.59 | + |
| MM48 | ENSMUSG00000055882 | ENSMUSP00000066520 | 2 | + | 181210265 | 181211686 | ENSMUSG00000007036 | ENSMUSP00000007251 | 17 | 0.02 | 14.93 | + |
| MM49 | ENSMUSG00000048550 | ENSMUSP00000052452 | 2 | + | 21253448 | 21255688 | ENSMUSG00000054474 | ENSMUSP00000073861 | 6 | 0.01 | 61.26 | + |
| MM50 | ENSMUSG00000048652 | ENSMUSP00000051453 | 3 | - | 145627026 | 145627703 | ENSMUSG00000029131 | ENSMUSP00000008733 | 5 | 0.16 | 3.73 | + |
| MM51 | ENSMUSG00000037737 | ENSMUSP00000048360 | 3 | - | 30007936 | 30008859 | ENSMUSG00000031972 | ENSMUSP00000034453 | 8 | 0.03 | 15.06 | + |
| MM52 | ENSMUSG00000044528 | ENSMUSP00000062635 | 3 | + | 123116358 | 123117446 | ENSMUSG00000025935 | ENSMUSP00000027068 | 1 | 0.13 | 2.27 | + |
| MM53 | - | - | 4 | - | 122349152 | 122350576 | ENSMUSG00000022186 | ENSMUSP00000022792 | 15 | 0.02 | 8.61 | + |
| MM54 | ENSMUSG00000047281 | ENSMUSP00000050374 | 4 | - | 132561941 | 132562696 | ENSMUSG00000022285 | ENSMUSP00000087624 | 15 | 0.00 | 76.61 | + |
| MM55 | ENSMUSG00000028310 | ENSMUSP00000029991 | 4 | - | 49597314 | 49597823 | ENSMUSG00000033953 | ENSMUSP00000020319 | 11 | 0.00 | 74.99 | + |
| MM56 | ENSMUSG00000056979 | ENSMUSP00000080575 | 4 | - | 56683553 | 56684806 | ENSMUSG00000062825 | ENSMUSP00000071486 | 11 | 0.01 | 82.00 | + |
| MM57 | ENSMUSG00000028287 | ENSMUSP00000029955 | 4 | + | 39589648 | 39590295 | ENSMUSG00000029430 | ENSMUSP00000031383 | 5 | 0.05 | 0.82 | - |
| MM58 | ENSMUSG00000035561 | ENSMUSP00000041260 | 4 | + | 45718387 | 45719943 | ENSMUSG00000029455 | ENSMUSP00000031411 | 5 | 0.02 | 8.12 | + |
| MM59 | ENSMUSG00000038165 | ENSMUSP00000042680 | 5 | + | 23111561 | 23112634 | ENSMUSG00000050627 | ENSMUSP00000061640 | 9 | 0.08 | 1.50 | - |
| MM60 | ENSMUSG00000038044 | ENSMUSP00000044932 | 5 | + | 23985655 | 23987340 | ENSMUSG00000025613 | ENSMUSP00000026704 | 16 | 0.01 | 59.20 | + |
| MM61 | ENSMUSG00000050180 | ENSMUSP00000062942 | 5 | + | 51178801 | 51179457 | ENSMUSG00000022982 | ENSMUSP00000023707 | 16 | 0.01 | 67.60 | + |
| MM62 | ENSMUSG00000029204 | ENSMUSP00000031106 | 5 | + | 64694046 | 64694648 | ENSMUSG00000001847 | ENSMUSP00000079380 | 5 | 0.01 | 71.19 | + |
| MM63 | ENSMUSG00000051695 | ENSMUSP00000054863 | 6 | - | 86959616 | 86960683 | ENSMUSG00000056851 | ENSMUSP00000076294 | 15 | 0.08 | 0.99 | + |
| MM64 | ENSMUSG00000048825 | ENSMUSP00000062845 | 6 | - | 96626565 | 96627884 | ENSMUSG00000016619 | ENSMUSP00000016763 | 15 | 0.35 | 0.80 | - |
| MM65 | ENSMUSG00000056197 | ENSMUSP00000068770 | 6 | - | 73802361 | 73803275 | ENSMUSG00000005078 | ENSMUSP00000061370 | 12 | 0.28 | 0.68 | - |
| MM66 | ENSMUSG00000041791 | ENSMUSP00000038562 | 6 | + | 140833715 | 140834611 | ENSMUSG00000015733 | ENSMUSP00000015877 | 6 | 0.05 | 13.22 | + |
| MM67 | ENSMUSG00000036463 | ENSMUSP00000045487 | 6 | + | 66210606 | 66211253 | ENSMUSG00000007815 | ENSMUSP00000007959 | 9 | 0.15 | 0.63 | - |
| MM68 | ENSMUSG00000049332 | ENSMUSP00000050380 | 7 | - | 96345631 | 96346203 | ENSMUSG00000001847 | ENSMUSP00000079380 | 5 | 0.00 | 66.83 | + |
| MM69 | ENSMUSG00000055643 | ENSMUSP00000062054 | 7 | - | 98240444 | 98241973 | ENSMUSG00000005312 | ENSMUSP00000075782 | 13 | 0.05 | 15.29 | - |
| MM70 | ENSMUSG00000055826 | ENSMUSP00000064132 | 7 | - | 19507534 | 19508226 | ENSMUSG00000029359 | ENSMUSP00000031304 | 5 | 0.06 | 4.12 | - |
| MM71 | ENSMUSG00000025519 | ENSMUSP00000002025 | 8 | + | 65620898 | 65622796 | ENSMUSG00000021957 | ENSMUSP00000022529 | 14 | 0.01 | 35.56 | + |
| MM72 | ENSMUSG00000050445 | ENSMUSP00000052989 | 9 | - | 121928804 | 121930303 | ENSMUSG00000017969 | ENSMUSP00000018113 | 2 | 0.02 | 34.94 | + |
| MM73 | ENSMUSG00000051062 | ENSMUSP00000052219 | 11 | - | 35591545 | 35592249 | ENSMUSG00000046865 | ENSMUSP00000037613 | 7 | 0.01 | 11.37 | + |
| MM74 | ENSMUSG00000037096 | ENSMUSP00000036413 | 3 | - | 78694984 | 78695427 | ENSMUSG00000036029 | ENSMUSP00000087669 | 3 | 0.01 | 0.93 | - |
| MM75 | ENSMUSG00000037910 | ENSMUSP00000043341 | 3 | - | 48200639 | 48200953 | ENSMUSG00000021131 | ENSMUSP00000021559 | 12 | 0.12 | 1.61 | - |
| MM76 | ENSMUSG00000038797 | ENSMUSP00000042321 | 7 | + | 74677510 | 74679183 | ENSMUSG00000063488 | ENSMUSP00000073988 | 9 | 0.04 | 8.91 | + |
| MM77 | ENSMUSG00000032425 | ENSMUSP00000034998 | 9 | + | 88930280 | 88932001 | ENSMUSG00000061030 | ENSMUSP00000075044 | 2 | 0.17 | 3.03 | und |
| MM78 | ENSMUSG00000048124 | - | 1 | - | 80714828 | 80716906 | ENSMUSG00000019715 | ENSMUSP00000019859 | 2 | 0.35 | 0.21 | - |
| MM79 | - | - | 1 | - | 72005823 | 72006176 | ENSMUSG00000031950 | ENSMUSP00000034428 | 8 | 0.12 | 0.13 | - |
| MM80 | ENSMUSG00000066669 | ENSMUSP00000083011 | 1 | + | 175853095 | 175854402 | ENSMUSG00000035086 | ENSMUSP00000040094 | 11 | 0.08 | 5.33 | + |
| MM81 | ENSMUSG00000043859 | ENSMUSP00000054359 | 10 | - | 72035236 | 72035775 | ENSMUSG00000024165 | ENSMUSP00000024981 | 17 | 0.21 | 0.29 | - |
| MM82 | - | - | 10 | + | 99089148 | 99090812 | ENSMUSG00000039774 | ENSMUSP00000045721 | 4 | 0.05 | 6.70 | + |
| MM83 | ENSMUSG00000058809 | ENSMUSP00000073606 | 11 | + | 41251575 | 41253284 | ENSMUSG00000025980 | ENSMUSP00000027123 | 1 | 0.17 | 0.03 | - |
| MM84 | ENSMUSG00000020870 | ENSMUSP00000021240 | 11 | + | 94563064 | 94563774 | ENSMUSG00000036241 | ENSMUSP00000038813 | 4 | 0.03 | 4.12 | - |
| MM85 | ENSMUSG00000062899 | - | 11 | - | 22681608 | 22681997 | ENSMUSG00000037563 | ENSMUSP00000047384 | 7 | 0.23 | 0.21 | - |
| MM86 | ENSMUSG00000046620 | ENSMUSP00000082544 | 12 | + | 48041582 | 48042057 | ENSMUSG00000003429 | ENSMUSP00000003521 | 7 | 0.16 | 0.23 | - |
| MM87 | ENSMUSG00000034435 | ENSMUSP00000037476 | 12 | - | 70393114 | 70394142 | ENSMUSG00000032328 | ENSMUSP00000034878 | 9 | 0.00 | 80.63 | + |
| MM88 | ENSMUSG00000066489 | ENSMUSP00000082495 | 12 | - | 54364059 | 54364493 | ENSMUSG00000060938 | ENSMUSP00000073175 | 11 | 0.20 | 0.19 | - |
| MM89 | ENSMUSG00000063130 | ENSMUSP00000076880 | 13 | - | 3688138 | 3688596 | ENSMUSG00000019370 | ENSMUSP00000019514 | 7 | 0.01 | 7.76 | + |
| MM90 | ENSMUSG00000055194 | ENSMUSP00000052086 | 13 | + | 107504193 | 107505320 | ENSMUSG00000029580 | ENSMUSP00000031564 | 5 | 0.00 | 14.92 | + |
| MM91 | ENSMUSG00000021721 | ENSMUSP00000022235 | 13 | + | 101660306 | 101661598 | ENSMUSG00000032259 | ENSMUSP00000034798 | 9 | 0.01 | 82.87 | + |
| MM92 | ENSMUSG00000037359 | ENSMUSP00000038961 | 13 | - | 20981097 | 20981483 | ENSMUSG00000061518 | ENSMUSP00000079944 | 1 | 0.00 | 0.03 | - |
| MM93 | ENSMUSG00000043157 | ENSMUSP00000055447 | 14 | + | 55845536 | 55846075 | ENSMUSG00000020440 | ENSMUSP00000020717 | 6 | 0.01 | 34.57 | + |
| MM94 | ENSMUSG00000068021 | ENSMUSP00000086384 | 16 | + | 91234287 | 91236455 | ENSMUSG00000011034 | ENSMUSP00000011178 | 5 | 0.01 | 61.37 | + |
| MM95 | ENSMUSG00000048087 | ENSMUSP00000052276 | 16 | - | 45039735 | 45041039 | ENSMUSG00000027597 | ENSMUSP00000061851 | 2 | 0.00 | 0.01 | - |
| MM96 | ENSMUSG00000051065 | ENSMUSP00000053260 | 16 | - | 27616045 | 27617307 | ENSMUSG00000042826 | ENSMUSP00000054576 | 11 | 0.19 | 3.75 | + |
| MM97 | - | - | 17 | - | 63142339 | 63142818 | ENSMUSG00000034729 | ENSMUSP00000084176 | 17 | 0.00 | 0.04 | - |
| MM98 | - | - | 17 | + | 84935076 | 84935960 | ENSMUSG00000050043 | ENSMUSP00000059582 | 2 | 0.00 | 0.02 | - |
| MM99 | - | - | 18 | + | 15199592 | 15199951 | ENSMUSG00000024414 | ENSMUSP00000025278 | 11 | 0.00 | 0.05 | - |
| MM100 | ENSMUSG00000033272 | ENSMUSP00000036081 | 18 | + | 36905885 | 36906856 | ENSMUSG00000027957 | ENSMUSP00000029569 | 3 | 0.02 | 54.14 | + |
| MM101 | ENSMUSG00000061684 | ENSMUSP00000074346 | 18 | - | 82691294 | 82691773 | ENSMUSG00000041453 | ENSMUSP00000041652 | 5 | 0.05 | 0.06 | - |
| MM102 | ENSMUSG00000024815 | ENSMUSP00000025727 | 19 | + | 29256453 | 29257145 | ENSMUSG00000000827 | ENSMUSP00000068888 | 2 | 0.23 | 1.37 | + |
| MM103 | ENSMUSG00000045104 | ENSMUSP00000052356 | 19 | + | 21175305 | 21176309 | ENSMUSG00000030246 | ENSMUSP00000032373 | 6 | 0.10 | 0.07 | - |
| MM104 | ENSMUSG00000059555 | ENSMUSP00000080548 | 2 | - | 25126775 | 25128052 | ENSMUSG00000009563 | ENSMUSP00000009707 | 2 | 0.01 | 61.38 | + |
| MM105 | ENSMUSG00000035796 | ENSMUSP00000045085 | 2 | + | 35272572 | 35273123 | ENSMUSG00000024026 | ENSMUSP00000024823 | 17 | 0.00 | 0.02 | - |
| MM106 | - | - | 2 | - | 104419064 | 104419540 | ENSMUSG00000032171 | ENSMUSP00000034689 | 9 | 0.15 | 0.18 | - |
| MM107 | - | - | 2 | - | 134276681 | 134277484 | ENSMUSG00000039795 | ENSMUSP00000037459 | 3 | 0.00 | 0.02 | - |
| MM108 | - | - | 2 | - | 91228326 | 91228634 | ENSMUSG00000059355 | ENSMUSP00000078697 | 8 | 0.15 | 0.23 | - |
| MM109 | ENSMUSG00000069106 | ENSMUSP00000088891 | 3 | - | 11014552 | 11015313 | ENSMUSG00000011752 | ENSMUSP00000011896 | 19 | 0.00 | 0.02 | - |
| MM110 | ENSMUSG00000045235 | ENSMUSP00000052779 | 3 | - | 103072228 | 103073415 | ENSMUSG00000026280 | ENSMUSP00000027502 | 1 | 0.05 | 6.94 | - |
| MM111 | ENSMUSG00000059807 | ENSMUSP00000071927 | 3 | + | 82761665 | 82762375 | ENSMUSG00000031594 | ENSMUSP00000034003 | 8 | 0.02 | 23.42 | + |
| MM112 | ENSMUSG00000047676 | ENSMUSP00000087466 | 3 | - | 149052327 | 149053223 | ENSMUSG00000032518 | ENSMUSP00000035105 | 9 | 0.07 | 0.02 | - |
| MM113 | ENSMUSG00000049139 | ENSMUSP00000060300 | 4 | - | 4455354 | 4455875 | ENSMUSG00000028788 | ENSMUSP00000030578 | 4 | 0.12 | 0.94 | - |
| MM114 | ENSMUSG00000052024 | ENSMUSP00000065813 | 5 | - | 137335738 | 137337027 | ENSMUSG00000031672 | ENSMUSP00000034097 | 8 | 0.08 | 0.03 | und |
| MM115 | ENSMUSG00000056506 | ENSMUSP00000068975 | 5 | + | 7187782 | 7189071 | ENSMUSG00000036752 | ENSMUSP00000042342 | 2 | 0.13 | 0.05 | - |
| MM116 | ENSMUSG00000024437 | ENSMUSP00000025307 | 5 | - | 148054156 | 148054908 | ENSMUSG00000052102 | ENSMUSP00000069081 | 18 | 0.10 | 0.07 | - |
| MM117 | ENSMUSG00000057412 | ENSMUSP00000074089 | 6 | - | 127955705 | 127956181 | ENSMUSG00000001175 | ENSMUSP00000001204 | 12 | 0.00 | 24.38 | - |
| MM118 | ENSMUSG00000060782 | ENSMUSP00000075207 | 6 | + | 78566741 | 78567973 | ENSMUSG00000025580 | ENSMUSP00000026667 | 11 | 0.13 | 0.10 | - |
| MM119 | ENSMUSG00000047104 | - | 6 | - | 136070544 | 136071104 | ENSMUSG00000032959 | ENSMUSP00000048425 | 5 | 0.17 | 0.73 | - |
| MM120 | - | - | 6 | - | 96494977 | 96495345 | ENSMUSG00000036438 | ENSMUSP00000048857 | 17 | 0.00 | 35.43 | - |
| MM121 | ENSMUSG00000047896 | ENSMUSP00000059877 | 6 | - | 46732389 | 46733789 | ENSMUSG00000039191 | ENSMUSP00000040694 | 5 | 0.00 | 0.01 | - |
| MM122 | ENSMUSG00000051397 | ENSMUSP00000060099 | 6 | - | 67786832 | 67787782 | ENSMUSG00000045394 | ENSMUSP00000061935 | 17 | 0.01 | 79.03 | und |
| MM123 | - | - | 7 | - | 40906219 | 40906779 | ENSMUSG00000068798 | ENSMUSP00000088174 | 3 | 0.08 | 0.04 | - |
| MM124 | ENSMUSG00000053490 | - | 8 | - | 64127471 | 64128853 | ENSMUSG00000031651 | ENSMUSP00000050267 | 8 | 0.01 | 66.32 | + |
| MM125 | ENSMUSG00000053490 | ENSMUSP00000040299 | 8 | - | 64114474 | 64115871 | ENSMUSG00000045409 | ENSMUSP00000084415 | 17 | 0.04 | 17.47 | + |
| MM126 | - | - | 8 | + | 126869845 | 126870279 | ENSMUSG00000063457 | ENSMUSP00000069004 | 10 | 0.12 | 0.46 | - |
| MM127 | ENSMUSG00000059422 | ENSMUSP00000074371 | 9 | - | 79021403 | 79022602 | ENSMUSG00000024726 | ENSMUSP00000025632 | 19 | 0.00 | 0.01 | - |
| MM128 | - | - | 1 | - | 175619204 | 175621063 | ENSMUSG00000025531 | ENSMUSP00000026607 | X | 0.16 | 1.39 | + |
| MM129 | ENSMUSG00000037015 | - | 1 | + | 119277946 | 119278995 | ENSMUSG00000031191 | ENSMUSP00000033534 | X | 0.02 | 2.68 | + |
| MM130 | ENSMUSG00000026063 | ENSMUSP00000027230 | 1 | + | 31495205 | 31495858 | ENSMUSG00000042433 | ENSMUSP00000048754 | X | 0.24 | 0.18 | - |
| MM131 | ENSMUSG00000060879 | ENSMUSP00000074840 | 12 | - | 63063498 | 63064139 | ENSMUSG00000008682 | ENSMUSP00000008826 | X | 0.01 | 1.49 | + |
| MM132 | - | - | 12 | + | 53969904 | 53970857 | ENSMUSG00000031432 | ENSMUSP00000033809 | X | 0.01 | 0.12 | - |
| MM133 | - | - | 18 | + | 67434836 | 67435432 | ENSMUSG00000031242 | ENSMUSP00000033594 | X | 0.00 | 11.80 | + |
| MM134 | ENSMUSG00000043858 | ENSMUSP00000056785 | 7 | + | 38903734 | 38905068 | ENSMUSG00000067315 | ENSMUSP00000084660 | X | 0.02 | 16.24 | + |
| MM135 | ENSMUSG00000055707 | ENSMUSP00000069077 | 8 | - | 69606508 | 69608103 | ENSMUSG00000036782 | ENSMUSP00000041190 | X | 0.01 | 61.16 | + |
| MM136 | - | - | 1 | - | 128142832 | 128144013 | ENSMUSG00000032401 | ENSMUSP00000034969 | 9 | 0.01 | 70.99 | + |
| MM137 | ENSMUSG00000061631 | ENSMUSP00000071789 | 1 | - | 65288169 | 65288819 | ENSMUSG00000037805 | ENSMUSP00000048469 | 17 | 0.00 | 0.03 | - |
| MM138 | ENSMUSG00000059535 | ENSMUSP00000080402 | 1 | + | 86129579 | 86129905 | ENSMUSG00000062997 | ENSMUSP00000079672 | 2 | 0.21 | 0.35 | - |
| MM139 | ENSMUSG00000067017 | ENSMUSP00000083930 | 1 | + | 105347248 | 105348105 | ENSMUSG00000015733 | ENSMUSP00000015877 | 6 | 0.02 | 4.81 | - |
| MM140 | ENSMUSG00000067579 | ENSMUSP00000085271 | 1 | + | 43483874 | 43484353 | ENSMUSG00000048758 | ENSMUSP00000080203 | 9 | 0.00 | 0.03 | - |
| MM141 | ENSMUSG00000054364 | ENSMUSP00000067013 | 12 | - | 7815161 | 7815760 | ENSMUSG00000007815 | ENSMUSP00000007959 | 9 | 0.00 | 58.13 | + |
| MM142 | - | - | 13 | - | 30426473 | 30427297 | ENSMUSG00000020413 | ENSMUSP00000020683 | 11 | 0.25 | 1.64 | + |
| MM143 | ENSMUSG00000067002 | ENSMUSP00000083898 | 17 | - | 64131715 | 64132065 | ENSMUSG00000062006 | ENSMUSP00000087722 | 3 | 0.00 | 0.07 | - |
| MM144 | ENSMUSG00000046173 | ENSMUSP00000050792 | 17 | - | 8308653 | 8310581 | ENSMUSG00000022283 | ENSMUSP00000001809 | 15 | 0.16 | 0.60 | - |
| MM145 | ENSMUSG00000059000 | ENSMUSP00000078373 | 17 | - | 30643314 | 30645203 | ENSMUSG00000061030 | ENSMUSP00000075044 | 2 | 0.00 | 84.39 | und |
| MM146 | ENSMUSG00000053740 | ENSMUSP00000070266 | 18 | + | 14758409 | 14758897 | ENSMUSG00000032215 | ENSMUSP00000034738 | 9 | 0.00 | 0.02 | - |
| MM147 | ENSMUSG00000059040 | ENSMUSP00000075513 | 18 | + | 48268433 | 48269815 | ENSMUSG00000063524 | ENSMUSP00000079727 | 4 | 0.00 | 0.02 | - |
| MM148 | ENSMUSG00000062336 | ENSMUSP00000076642 | 19 | - | 44997748 | 44998632 | ENSMUSG00000029614 | ENSMUSP00000031617 | 5 | 0.15 | 0.05 | - |
| MM149 | ENSMUSG00000064193 | - | 2 | + | 80534822 | 80536192 | ENSMUSG00000063524 | ENSMUSP00000079727 | 4 | 0.00 | 0.02 | und |
| MM150 | - | - | 2 | - | 51803290 | 51803829 | ENSMUSG00000056954 | ENSMUSP00000003464 | 11 | 0.00 | 0.03 | - |
| MM151 | - | - | 2 | - | 116041695 | 116042339 | ENSMUSG00000000711 | ENSMUSP00000000727 | 10 | 0.00 | 0.02 | - |
| MM152 | ENSMUSG00000043155 | ENSMUSP00000062327 | 4 | - | 115779056 | 115780168 | ENSMUSG00000029445 | ENSMUSP00000031398 | 5 | 0.01 | 65.59 | + |
| MM153 | ENSMUSG00000059343 | ENSMUSP00000077687 | 4 | - | 71942507 | 71943598 | ENSMUSG00000030695 | ENSMUSP00000032934 | 7 | 0.14 | 0.18 | - |
| MM154 | ENSMUSG00000059195 | - | 4 | + | 102522305 | 102523432 | ENSMUSG00000062825 | ENSMUSP00000071486 | 11 | 0.00 | 0.02 | - |
| MM155 | - | - | 4 | + | 122143525 | 122144949 | ENSMUSG00000022186 | ENSMUSP00000022792 | 15 | 0.02 | 7.55 | + |
| MM156 | - | - | 4 | + | 143502494 | 143503879 | ENSMUSG00000037570 | ENSMUSP00000043901 | 15 | 0.11 | 0.02 | - |
| MM157 | ENSMUSG00000039899 | ENSMUSP00000046131 | 5 | + | 19827382 | 19828047 | ENSMUSG00000031594 | ENSMUSP00000034003 | 8 | 0.06 | 8.45 | + |
| MM158 | ENSMUSG00000066921 | ENSMUSP00000083694 | 5 | - | 114915797 | 114916276 | ENSMUSG00000048758 | ENSMUSP00000080203 | 9 | 0.00 | 0.03 | - |
| MM159 | ENSMUSG00000063047 | - | 7 | - | 23378713 | 23381417 | ENSMUSG00000038686 | ENSMUSP00000036627 | 6 | 0.30 | 1.19 | + |
| MM160 | ENSMUSG00000062040 | ENSMUSP00000054012 | 7 | - | 25308674 | 25311211 | ENSMUSG00000066021 | ENSMUSP00000070028 | 4 | 0.11 | 4.00 | + |
| MM161 | ENSMUSG00000058402 | ENSMUSP00000056077 | 7 | + | 25288877 | 25290871 | ENSMUSG00000061030 | ENSMUSP00000075044 | 2 | 0.00 | 87.26 | + |
| MM162 | ENSMUSG00000034308 | ENSMUSP00000044457 | 8 | - | 116996625 | 116997731 | ENSMUSG00000035077 | ENSMUSP00000081683 | 7 | 0.04 | 11.46 | + |
| MM163 | - | - | 8 | - | 40598695 | 40599717 | ENSMUSG00000037935 | ENSMUSP00000036851 | 11 | 0.17 | 0.05 | - |

^1^ ENSG and ENSP are Ensembl identifiers for genes and proteins, respectively.

^2^ “Human shared” indicates whether the dating procedure detects an orthologous retrogene in the human genome. ‘und’ stands for undefined.
