# Supplementary material for: Chromosomal Gene Movements Reflect the Recent Origin and Biology of Therian Sex Chromosomes
Source: PLoS Biol. 2008 Apr 1;6(4):e80. doi: 10.1371/journal.pbio.0060080 (PMC2276528; doi:10.1371/journal.pbio.0060080)
Supplement: Supplementary file 4 [file pbio.0060080.st003.doc]

**Table S3.** Dog retrogene set.

| Retro. ID | Retro. ENSG^1^ | Retro. ENSP^1^ | Retro. chr. | Retro. strand | Retro. start | Retro. end | Par. ENSG^1^ | Par. ENSP^1^ | Par. chr. | *d*_N_/*d*_S_ | *d*_S_ | Human shared^2^ |
| --- | --- | --- | --- | --- | --- | --- | --- | --- | --- | --- | --- | --- |
| CF1 | ENSCAFG00000001926 | ENSCAFP00000002831 | 1 | - | 91237253 | 91238086 | ENSCAFG00000018882 | ENSCAFP00000027852 | X | 0.09 | 2.08 | + |
| CF2 | ENSCAFG00000000371 | ENSCAFP00000000524 | 1 | + | 42627255 | 42627848 | ENSCAFG00000017383 | ENSCAFP00000025615 | X | 0.09 | 6.64 | - |
| CF3 | ENSCAFG00000001594 | ENSCAFP00000002316 | 11 | + | 40237114 | 40238055 | ENSCAFG00000016503 | ENSCAFP00000024281 | X | 0.00 | 2.10 | + |
| CF4 | ENSCAFG00000001662 | ENSCAFP00000002421 | 11 | + | 42148631 | 42150592 | ENSCAFG00000018272 | ENSCAFP00000026997 | X | 0.05 | 0.78 | + |
| CF5 | ENSCAFG00000002146 | ENSCAFP00000003135 | 12 | - | 20554657 | 20555907 | ENSCAFG00000017270 | ENSCAFP00000025449 | X | 0.10 | 0.73 | + |
| CF6 | ENSCAFG00000004850 | ENSCAFP00000007240 | 19 | + | 32931996 | 32933045 | ENSCAFG00000019103 | ENSCAFP00000028179 | X | 0.04 | 1.28 | + |
| CF7 | ENSCAFG00000005346 | ENSCAFP00000007975 | 2 | + | 31307135 | 31307677 | ENSCAFG00000018545 | ENSCAFP00000027372 | X | 0.00 | 10.35 | - |
| CF8 | - | - | 21 | + | 45447035 | 45448186 | ENSCAFG00000019286 | ENSCAFP00000028461 | X | 0.14 | 0.98 | + |
| CF9 | ENSCAFG00000005152 | ENSCAFP00000007695 | 24 | - | 3300892 | 3301332 | ENSCAFG00000018045 | ENSCAFP00000026668 | X | 0.11 | 1.42 | + |
| CF10 | ENSCAFG00000006653 | ENSCAFP00000009940 | 27 | - | 4478796 | 4480646 | ENSCAFG00000016908 | ENSCAFP00000024875 | X | 0.33 | 0.58 | - |
| CF11 | - | - | 28 | + | 32976287 | 32976565 | ENSCAFG00000019146 | ENSCAFP00000028244 | X | 0.02 | 27.56 | + |
| CF12 | ENSCAFG00000008862 | ENSCAFP00000013015 | 32 | - | 6617728 | 6618414 | ENSCAFG00000019415 | ENSCAFP00000028702 | X | 0.07 | 1.23 | + |
| CF13 | ENSCAFG00000008864 | ENSCAFP00000013019 | 32 | - | 6692439 | 6694103 | ENSCAFG00000013713 | ENSCAFP00000020225 | X | 0.19 | 0.42 | + |
| CF14 | ENSCAFG00000019978 | ENSCAFP00000029600 | 5 | + | 73114090 | 73115193 | ENSCAFG00000019172 | ENSCAFP00000028277 | X | 0.08 | 9.18 | + |
| CF15 | ENSCAFG00000019792 | ENSCAFP00000029296 | 6 | - | 43408632 | 43409105 | ENSCAFG00000019590 | ENSCAFP00000028970 | X | 0.01 | 79.92 | + |
| CF16 | - | - | 7 | - | 35652976 | 35654925 | ENSCAFG00000017406 | ENSCAFP00000025660 | X | 0.23 | 0.77 | + |
| CF17 | ENSCAFG00000018336 | ENSCAFP00000027088 | 7 | + | 69902659 | 69903177 | ENSCAFG00000019170 | ENSCAFP00000028275 | X | 0.02 | 4.79 | + |
| CF18 | ENSCAFG00000014147 | ENSCAFP00000020851 | 8 | - | 26546443 | 26547093 | ENSCAFG00000019553 | ENSCAFP00000028918 | X | 0.00 | 0.61 | + |
| CF19 | ENSCAFG00000020192 | ENSCAFP00000029943 | 9 | - | 50407570 | 50408196 | ENSCAFG00000018870 | ENSCAFP00000027831 | X | 0.14 | 0.27 | - |
| CF20 | - | - | 1 | - | 49885499 | 49886488 | ENSCAFG00000009211 | ENSCAFP00000013575 | 21 | 0.15 | 1.17 | + |
| CF21 | ENSCAFG00000001109 | ENSCAFP00000001565 | 1 | - | 70736834 | 70737661 | ENSCAFG00000019317 | ENSCAFP00000028536 | 6 | 0.21 | 0.09 | - |
| CF22 | ENSCAFG00000004697 | ENSCAFP00000007004 | 1 | - | 113418596 | 113420182 | ENSCAFG00000004680 | ENSCAFP00000006968 | 1 | 0.18 | 1.96 | + |
| CF23 | ENSCAFG00000000021 | ENSCAFP00000000032 | 1 | + | 7182017 | 7182952 | ENSCAFG00000003963 | ENSCAFP00000005896 | 17 | 0.02 | 57.64 | + |
| CF24 | - | - | 1 | + | 111976924 | 111978087 | ENSCAFG00000017158 | ENSCAFP00000025272 | 8 | 0.00 | 80.60 | + |
| CF25 | ENSCAFG00000007214 | ENSCAFP00000010717 | 1 | + | 119888734 | 119889738 | ENSCAFG00000019757 | ENSCAFP00000029243 | 9 | 0.11 | 1.66 | - |
| CF26 | ENSCAFG00000001137 | ENSCAFP00000001600 | 10 | + | 27537513 | 27538439 | ENSCAFG00000005174 | ENSCAFP00000007728 | 16 | 0.29 | 1.01 | + |
| CF27 | ENSCAFG00000002720 | ENSCAFP00000003985 | 10 | + | 57544612 | 57545157 | ENSCAFG00000008778 | ENSCAFP00000012918 | 27 | 0.02 | 1.67 | - |
| CF28 | ENSCAFG00000001143 | ENSCAFP00000001617 | 10 | - | 27693323 | 27694312 | ENSCAFG00000003533 | ENSCAFP00000005250 | 12 | 0.04 | 14.37 | + |
| CF29 | ENSCAFG00000002630 | ENSCAFP00000003823 | 10 | - | 52005269 | 52006015 | ENSCAFG00000016151 | ENSCAFP00000023805 | 27 | 0.16 | 1.03 | + |
| CF30 | ENSCAFG00000002400 | ENSCAFP00000003484 | 11 | + | 55927883 | 55929382 | ENSCAFG00000008683 | ENSCAFP00000012800 | 26 | 0.03 | 5.30 | + |
| CF31 | ENSCAFG00000000154 | ENSCAFP00000000223 | 11 | + | 3011726 | 3012346 | ENSCAFG00000015901 | ENSCAFP00000023396 | 18 | 0.04 | 3.32 | + |
| CF32 | ENSCAFG00000001357 | ENSCAFP00000001946 | 11 | - | 28431359 | 28434628 | ENSCAFG00000005561 | ENSCAFP00000008328 | 22 | 0.16 | 0.71 | + |
| CF33 | ENSCAFG00000001132 | ENSCAFP00000001592 | 11 | - | 26837184 | 26838059 | ENSCAFG00000006580 | ENSCAFP00000009851 | 27 | 0.02 | 21.99 | + |
| CF34 | - | - | 11 | - | 59656889 | 59657395 | ENSCAFG00000003248 | ENSCAFP00000004831 | 10 | 0.02 | 4.48 | + |
| CF35 | ENSCAFG00000002205 | ENSCAFP00000003212 | 12 | + | 22989853 | 22990917 | ENSCAFG00000017523 | ENSCAFP00000025812 | 30 | 0.11 | 6.88 | + |
| CF36 | - | - | 12 | + | 19060507 | 19061568 | ENSCAFG00000003028 | ENSCAFP00000004521 | 11 | 0.00 | 0.01 | - |
| CF37 | ENSCAFG00000001575 | ENSCAFP00000002291 | 12 | + | 12746014 | 12746553 | ENSCAFG00000013897 | ENSCAFP00000020480 | 27 | 0.04 | 16.21 | + |
| CF38 | ENSCAFG00000004062 | ENSCAFP00000006028 | 12 | - | 74781448 | 74782566 | ENSCAFG00000007120 | ENSCAFP00000010560 | 25 | 0.18 | 3.27 | + |
| CF39 | ENSCAFG00000003564 | ENSCAFP00000005295 | 12 | - | 61992906 | 61994114 | ENSCAFG00000001346 | ENSCAFP00000001940 | 10 | 0.04 | 0.03 | - |
| CF40 | ENSCAFG00000002867 | ENSCAFP00000004240 | 13 | - | 62362660 | 62363181 | ENSCAFG00000018505 | ENSCAFP00000027321 | 7 | 0.18 | 0.18 | - |
| CF41 | ENSCAFG00000001012 | ENSCAFP00000001431 | 13 | - | 24985563 | 24986945 | ENSCAFG00000003916 | ENSCAFP00000005831 | 17 | 0.01 | 95.95 | + |
| CF42 | ENSCAFG00000001530 | ENSCAFP00000002207 | 14 | + | 10067543 | 10067965 | ENSCAFG00000013366 | ENSCAFP00000019699 | 36 | 0.00 | 56.46 | - |
| CF43 | - | - | 14 | + | 3929497 | 3929700 | ENSCAFG00000017321 | ENSCAFP00000025525 | 20 | 0.00 | 0.07 | - |
| CF44 | - | - | 14 | + | 39968506 | 39968826 | ENSCAFG00000003257 | ENSCAFP00000004838 | 17 | 0.17 | 0.75 | - |
| CF45 | ENSCAFG00000002440 | ENSCAFP00000003561 | 14 | + | 34188162 | 34189112 | ENSCAFG00000001290 | ENSCAFP00000001850 | 14 | 0.00 | 0.01 | - |
| CF46 | ENSCAFG00000002263 | ENSCAFP00000003292 | 14 | + | 28368715 | 28369275 | ENSCAFG00000019044 | ENSCAFP00000028105 | 7 | 0.00 | 0.03 | - |
| CF47 | ENSCAFG00000001464 | ENSCAFP00000002117 | 14 | - | 9069876 | 9070151 | ENSCAFG00000014461 | ENSCAFP00000021343 | 37 | 0.05 | 0.09 | - |
| CF48 | ENSCAFG00000003337 | ENSCAFP00000004940 | 14 | - | 55135268 | 55136377 | ENSCAFG00000010964 | ENSCAFP00000016154 | 25 | 0.02 | 57.56 | + |
| CF49 | ENSCAFG00000002312 | ENSCAFP00000003353 | 14 | - | 29925012 | 29925287 | ENSCAFG00000014461 | ENSCAFP00000021343 | 37 | 0.06 | 0.94 | - |
| CF50 | ENSCAFG00000004619 | ENSCAFP00000006895 | 15 | - | 18009413 | 18010528 | ENSCAFG00000008093 | ENSCAFP00000011984 | 26 | 0.01 | 70.50 | + |
| CF51 | ENSCAFG00000003671 | ENSCAFP00000005459 | 15 | - | 10917032 | 10917601 | ENSCAFG00000007851 | ENSCAFP00000011606 | 25 | 0.07 | 5.90 | + |
| CF52 | ENSCAFG00000008440 | ENSCAFP00000012399 | 15 | - | 55269549 | 55270247 | ENSCAFG00000007021 | ENSCAFP00000010427 | 16 | 0.04 | 13.34 | + |
| CF53 | ENSCAFG00000006525 | ENSCAFP00000009766 | 16 | + | 36016636 | 36017607 | ENSCAFG00000003348 | ENSCAFP00000004977 | 17 | 0.03 | 29.97 | + |
| CF54 | ENSCAFG00000004961 | ENSCAFP00000007378 | 16 | + | 18093309 | 18094868 | ENSCAFG00000008687 | ENSCAFP00000012804 | 31 | 0.01 | 58.56 | + |
| CF55 | ENSCAFG00000003165 | ENSCAFP00000004723 | 16 | + | 4527756 | 4528391 | ENSCAFG00000009062 | ENSCAFP00000013328 | 28 | 0.07 | 4.95 | + |
| CF56 | - | - | 17 | + | 40505280 | 40506140 | ENSCAFG00000007306 | ENSCAFP00000010844 | 24 | 0.22 | 0.29 | - |
| CF57 | ENSCAFG00000006297 | ENSCAFP00000009456 | 17 | + | 33685977 | 33687641 | ENSCAFG00000001545 | ENSCAFP00000002233 | 11 | 0.25 | 1.86 | + |
| CF58 | ENSCAFG00000005126 | ENSCAFP00000007639 | 19 | - | 41635447 | 41636655 | ENSCAFG00000017322 | ENSCAFP00000025530 | 30 | 0.01 | 71.20 | + |
| CF59 | ENSCAFG00000006961 | ENSCAFP00000010347 | 2 | - | 45127410 | 45128558 | ENSCAFG00000008253 | ENSCAFP00000012116 | 30 | 0.02 | 2.90 | + |
| CF60 | ENSCAFG00000007276 | ENSCAFP00000010789 | 2 | - | 50623461 | 50624729 | ENSCAFG00000013890 | ENSCAFP00000020469 | 5 | 0.00 | 118.91 | + |
| CF61 | ENSCAFG00000009413 | ENSCAFP00000013860 | 2 | - | 61056176 | 61056802 | ENSCAFG00000006584 | ENSCAFP00000009845 | 1 | 0.05 | 3.30 | + |
| CF62 | ENSCAFG00000012290 | ENSCAFP00000018103 | 2 | - | 73930300 | 73931040 | ENSCAFG00000003386 | ENSCAFP00000005031 | 17 | 0.01 | 13.44 | + |
| CF63 | ENSCAFG00000006109 | ENSCAFP00000009182 | 2 | - | 36887370 | 36888299 | ENSCAFG00000005955 | ENSCAFP00000008926 | 25 | 0.14 | 0.62 | + |
| CF64 | ENSCAFG00000004265 | ENSCAFP00000006336 | 2 | + | 17258780 | 17261005 | ENSCAFG00000007408 | ENSCAFP00000011010 | 17 | 0.01 | 60.28 | + |
| CF65 | ENSCAFG00000005235 | ENSCAFP00000007824 | 2 | + | 31024664 | 31025113 | ENSCAFG00000002646 | ENSCAFP00000003862 | 10 | 0.01 | 61.10 | + |
| CF66 | ENSCAFG00000004138 | ENSCAFP00000006139 | 20 | - | 5288958 | 5289656 | ENSCAFG00000005174 | ENSCAFP00000007728 | 16 | 0.00 | 73.42 | + |
| CF67 | - | - | 21 | - | 46995257 | 46995634 | ENSCAFG00000005709 | ENSCAFP00000008545 | 2 | 0.02 | 1.24 | - |
| CF68 | ENSCAFG00000004440 | ENSCAFP00000006620 | 21 | - | 16128193 | 16128711 | ENSCAFG00000002490 | ENSCAFP00000003626 | 12 | 0.24 | 0.48 | + |
| CF69 | ENSCAFG00000004848 | ENSCAFP00000007234 | 21 | + | 23832199 | 23832861 | ENSCAFG00000010773 | ENSCAFP00000015860 | 6 | 0.01 | 77.67 | + |
| CF70 | ENSCAFG00000005289 | ENSCAFP00000007899 | 22 | - | 40864579 | 40867113 | ENSCAFG00000005293 | ENSCAFP00000007910 | 22 | 0.01 | 63.29 | + |
| CF71 | ENSCAFG00000005370 | ENSCAFP00000008003 | 23 | - | 14978403 | 14979902 | ENSCAFG00000011426 | ENSCAFP00000016804 | 24 | 0.04 | 12.26 | + |
| CF72 | ENSCAFG00000011481 | ENSCAFP00000016866 | 24 | + | 39357502 | 39358071 | ENSCAFG00000005582 | ENSCAFP00000008345 | 1 | 0.23 | 0.55 | und |
| CF73 | ENSCAFG00000009338 | ENSCAFP00000013759 | 24 | - | 34326993 | 34327448 | ENSCAFG00000006463 | ENSCAFP00000009674 | 27 | 0.25 | 1.64 | + |
| CF74 | ENSCAFG00000011889 | ENSCAFP00000017497 | 25 | - | 48186600 | 48187313 | ENSCAFG00000005174 | ENSCAFP00000007728 | 16 | 0.03 | 4.87 | + |
| CF75 | ENSCAFG00000013594 | ENSCAFP00000020005 | 26 | - | 29289559 | 29291166 | ENSCAFG00000016112 | ENSCAFP00000023797 | 30 | 0.33 | 0.82 | + |
| CF76 | - | - | 26 | + | 12600220 | 12602391 | ENSCAFG00000005847 | ENSCAFP00000008762 | 16 | 0.02 | 27.66 | + |
| CF77 | ENSCAFG00000009028 | ENSCAFP00000013266 | 27 | + | 9618933 | 9619265 | ENSCAFG00000002398 | ENSCAFP00000003483 | 10 | 0.03 | 23.93 | + |
| CF78 | ENSCAFG00000013245 | ENSCAFP00000019506 | 27 | + | 36679773 | 36681017 | ENSCAFG00000000015 | ENSCAFP00000000020 | 1 | 0.03 | 26.79 | + |
| CF79 | ENSCAFG00000012506 | ENSCAFP00000018414 | 27 | - | 31370151 | 31371047 | ENSCAFG00000003408 | ENSCAFP00000005070 | 14 | 0.05 | 11.19 | + |
| CF80 | ENSCAFG00000007044 | ENSCAFP00000010469 | 28 | + | 6679881 | 6681125 | ENSCAFG00000000336 | ENSCAFP00000000470 | 11 | 0.12 | 1.00 | + |
| CF81 | ENSCAFG00000007080 | ENSCAFP00000010515 | 29 | - | 11242542 | 11242817 | ENSCAFG00000014461 | ENSCAFP00000021343 | 37 | 0.19 | 0.89 | - |
| CF82 | ENSCAFG00000015116 | ENSCAFP00000022279 | 3 | + | 66189389 | 66190447 | ENSCAFG00000008253 | ENSCAFP00000012116 | 30 | 0.03 | 1.46 | - |
| CF83 | ENSCAFG00000016498 | ENSCAFP00000024271 | 3 | - | 88570375 | 88570782 | ENSCAFG00000008859 | ENSCAFP00000013012 | 31 | 0.01 | 71.12 | + |
| CF84 | ENSCAFG00000015923 | ENSCAFP00000023421 | 3 | - | 75455441 | 75456031 | ENSCAFG00000013413 | ENSCAFP00000019762 | 17 | 0.01 | 84.76 | + |
| CF85 | ENSCAFG00000010679 | ENSCAFP00000015703 | 3 | - | 43341711 | 43341944 | ENSCAFG00000014258 | ENSCAFP00000021038 | und | 0.04 | 13.39 | + |
| CF86 | - | - | 3 | - | 39368862 | 39369914 | ENSCAFG00000003953 | ENSCAFP00000005883 | 16 | 0.24 | 1.09 | + |
| CF87 | ENSCAFG00000012037 | ENSCAFP00000017722 | 30 | - | 13098624 | 13100678 | ENSCAFG00000017485 | ENSCAFP00000025766 | 6 | 0.01 | 64.05 | + |
| CF88 | ENSCAFG00000009406 | ENSCAFP00000013853 | 32 | + | 12957238 | 12958131 | ENSCAFG00000017274 | ENSCAFP00000025446 | 4 | 0.00 | 0.02 | - |
| CF89 | ENSCAFG00000011263 | ENSCAFP00000016569 | 32 | + | 31752272 | 31752610 | ENSCAFG00000005119 | ENSCAFP00000007624 | 23 | 0.02 | 2.96 | - |
| CF90 | - | - | 32 | - | 6097197 | 6097469 | ENSCAFG00000017253 | ENSCAFP00000025406 | 20 | 0.16 | 1.20 | - |
| CF91 | - | - | 32 | - | 39513562 | 39514026 | ENSCAFG00000007866 | ENSCAFP00000011621 | 29 | 0.20 | 1.09 | + |
| CF92 | ENSCAFG00000011894 | ENSCAFP00000017508 | 33 | - | 28627233 | 28628219 | ENSCAFG00000019830 | ENSCAFP00000029367 | 9 | 0.08 | 1.27 | + |
| CF93 | - | - | 33 | + | 34434929 | 34435318 | ENSCAFG00000000451 | ENSCAFP00000000668 | 10 | 0.01 | 73.65 | + |
| CF94 | - | - | 33 | + | 34452969 | 34453358 | ENSCAFG00000000451 | ENSCAFP00000000668 | 10 | 0.17 | 5.36 | + |
| CF95 | ENSCAFG00000014677 | ENSCAFP00000021650 | 34 | - | 37451115 | 37452065 | ENSCAFG00000008253 | ENSCAFP00000012116 | 30 | 0.02 | 22.93 | + |
| CF96 | ENSCAFG00000011235 | ENSCAFP00000016613 | 35 | - | 27754272 | 27756529 | ENSCAFG00000000299 | ENSCAFP00000000442 | 11 | 0.21 | 1.60 | + |
| CF97 | ENSCAFG00000019051 | ENSCAFP00000028113 | 4 | - | 89330679 | 89331599 | ENSCAFG00000015830 | ENSCAFP00000023272 | 5 | 0.04 | 4.22 | - |
| CF98 | ENSCAFG00000016400 | ENSCAFP00000024132 | 4 | + | 39160345 | 39160752 | ENSCAFG00000008383 | ENSCAFP00000012313 | 23 | 0.01 | 57.36 | + |
| CF99 | - | - | 5 | - | 30954455 | 30955642 | ENSCAFG00000016220 | ENSCAFP00000023905 | 2 | 0.11 | 1.29 | + |
| CF100 | ENSCAFG00000016612 | ENSCAFP00000024451 | 6 | - | 18693828 | 18696601 | ENSCAFG00000005947 | ENSCAFP00000008942 | 26 | 0.04 | 11.82 | + |
| CF101 | ENSCAFG00000015763 | ENSCAFP00000023183 | 7 | + | 35945650 | 35946954 | ENSCAFG00000014744 | ENSCAFP00000021764 | 9 | 0.37 | 1.08 | + |
| CF102 | - | - | 7 | + | 79977854 | 79978414 | ENSCAFG00000019822 | ENSCAFP00000029352 | 5 | 0.04 | 8.68 | + |
| CF103 | ENSCAFG00000018730 | ENSCAFP00000027629 | 7 | + | 78300490 | 78300825 | ENSCAFG00000002906 | ENSCAFP00000004307 | 11 | 0.11 | 2.21 | + |
| CF104 | ENSCAFG00000016313 | ENSCAFP00000024032 | 7 | + | 42259169 | 42259843 | ENSCAFG00000006580 | ENSCAFP00000009851 | 27 | 0.18 | 0.20 | - |
| CF105 | - | - | 8 | + | 6870603 | 6870818 | ENSCAFG00000012676 | ENSCAFP00000018663 | 24 | 0.20 | 1.34 | - |
| CF106 | ENSCAFG00000011311 | ENSCAFP00000016648 | 8 | + | 6214910 | 6215590 | ENSCAFG00000000829 | ENSCAFP00000001185 | 12 | 0.01 | 78.29 | + |
| CF107 | ENSCAFG00000016031 | ENSCAFP00000023616 | 8 | + | 41870954 | 41872864 | ENSCAFG00000011666 | ENSCAFP00000017166 | 5 | 0.00 | 16.30 | + |
| CF108 | ENSCAFG00000014231 | ENSCAFP00000020964 | 8 | + | 29320004 | 29320549 | ENSCAFG00000008778 | ENSCAFP00000012918 | 27 | 0.00 | 48.79 | + |
| CF109 | ENSCAFG00000019127 | ENSCAFP00000028225 | 9 | - | 37640419 | 37640733 | ENSCAFG00000004895 | ENSCAFP00000007299 | 19 | 0.01 | 78.89 | + |
| CF110 | ENSCAFG00000020165 | ENSCAFP00000029904 | 9 | - | 48962277 | 48963800 | ENSCAFG00000014502 | ENSCAFP00000021401 | 7 | 0.01 | 48.15 | + |
| CF111 | ENSCAFG00000017563 | ENSCAFP00000025887 | 9 | - | 26005231 | 26005716 | ENSCAFG00000017516 | ENSCAFP00000025799 | 8 | 0.00 | 5.86 | - |
| CF112 | ENSCAFG00000017331 | ENSCAFP00000025537 | 9 | + | 21885771 | 21887474 | ENSCAFG00000007191 | ENSCAFP00000010694 | 2 | 0.01 | 18.47 | + |

^1^ ENSG and ENSP are Ensembl identifiers for genes and proteins, respectively.

^2^ “Human shared” indicates whether the dating procedure detects an orthologous retrogene in the human genome. ‘und’ stands for undefined.
