# Supplementary material for: Chromosomal Gene Movements Reflect the Recent Origin and Biology of Therian Sex Chromosomes
Source: PLoS Biol. 2008 Apr 1;6(4):e80. doi: 10.1371/journal.pbio.0060080 (PMC2276528; doi:10.1371/journal.pbio.0060080)
Supplement: Supplementary file 5 [file pbio.0060080.st004.doc]

**Table S4.** Opossum retrogene set.

| Retro. ID | Retro. ENSG^1^ | Retro. ENSP^1^ | Retro. chr. | Retro. strand | Retro. start | Retro. end | Par. ENSG^1^ | Par. ENSP^1^ | Par. chr. | *d*_N_/*d*_S_ | *d*_S_ | Human shared^2^ |
| --- | --- | --- | --- | --- | --- | --- | --- | --- | --- | --- | --- | --- |
| MD1 | ENSMODG00000002836 | ENSMODP00000030940 | 1 | - | 387190811 | 387191452 | ENSMODG00000015178 | ENSMODP00000018964 | X | 0.05 | 0.76 | - |
| MD2 | ENSMODG00000000199 | ENSMODP00000000232 | 1 | - | 454861715 | 454863055 | ENSMODG00000014087 | ENSMODP00000017614 | X | 0.17 | 0.74 | - |
| MD3 | - | - | 1 | + | 530390635 | 530390925 | ENSMODG00000005753 | ENSMODP00000007127 | X | 0.18 | 0.65 | - |
| MD4 | ENSMODG00000001710 | ENSMODP00000002095 | 2 | + | 479488259 | 479488789 | ENSMODG00000008997 | ENSMODP00000011222 | X | 0.01 | 31.80 | + |
| MD5 | ENSMODG00000023304 | ENSMODP00000029378 | 2 | - | 414142649 | 414143899 | ENSMODG00000004055 | ENSMODP00000004988 | X | 0.15 | 0.17 | - |
| MD6 | ENSMODG00000023385 | ENSMODP00000029718 | 2 | - | 355842995 | 355844245 | ENSMODG00000004055 | ENSMODP00000004988 | X | 0.11 | 0.77 | - |
| MD7 | - | - | 3 | - | 106822165 | 106823334 | ENSMODG00000002312 | ENSMODP00000002817 | X | 0.24 | 0.29 | - |
| MD8 | ENSMODG00000005318 | ENSMODP00000006555 | 3 | + | 143752114 | 143752644 | ENSMODG00000009181 | ENSMODP00000011455 | X | 0.06 | 0.72 | - |
| MD9 | ENSMODG00000025382 | ENSMODP00000037958 | 3 | + | 19315790 | 19316305 | ENSMODG00000005932 | ENSMODP00000007336 | X | 0.19 | 0.33 | - |
| MD10 | ENSMODG00000024803 | ENSMODP00000035687 | 4 | + | 177754478 | 177755197 | ENSMODG00000008300 | ENSMODP00000010317 | X | 0.06 | 5.38 | - |
| MD11 | - | - | 4 | - | 369337557 | 369338861 | ENSMODG00000008869 | ENSMODP00000011047 | X | 0.26 | 1.16 | - |
| MD12 | ENSMODG00000025017 | ENSMODP00000036595 | 5 | - | 101156242 | 101157492 | ENSMODG00000004055 | ENSMODP00000004988 | X | 0.13 | 0.09 | - |
| MD13 | ENSMODG00000025085 | ENSMODP00000036820 | 5 | + | 80613541 | 80614134 | ENSMODG00000004273 | ENSMODP00000005264 | X | 0.00 | 5.92 | - |
| MD14 | ENSMODG00000025206 | ENSMODP00000037363 | 6 | - | 29637143 | 29637808 | ENSMODG00000004273 | ENSMODP00000005264 | X | 0.15 | 0.27 | - |
| MD15 | - | - | 6 | + | 238144063 | 238144848 | ENSMODG00000008851 | ENSMODP00000011025 | X | 0.00 | 56.11 | - |
| MD16 | - | - | 7 | - | 58602606 | 58604510 | ENSMODG00000014364 | ENSMODP00000017951 | X | 0.26 | 0.36 | - |
| MD17 | - | - | 8 | - | 126035584 | 126036186 | ENSMODG00000011382 | ENSMODP00000014236 | X | 0.12 | 1.80 | - |
| MD18 | - | - | 8 | - | 176432663 | 176433637 | ENSMODG00000002362 | ENSMODP00000002876 | X | 0.10 | 3.93 | + |
| MD19 | - | - | 8 | + | 258686112 | 258687866 | ENSMODG00000005195 | ENSMODP00000006397 | X | 0.19 | 1.44 | - |
| MD20 | - | - | 8 | + | 28344642 | 28345142 | ENSMODG00000003863 | ENSMODP00000004731 | X | 0.02 | 3.04 | - |
| MD21 | - | - | 1 | + | 699507741 | 699509363 | ENSMODG00000014967 | ENSMODP00000018716 | 6 | 0.25 | 1.77 | + |
| MD22 | ENSMODG00000002041 | ENSMODP00000002489 | 1 | - | 638270916 | 638271851 | ENSMODG00000023818 | ENSMODP00000031775 | 1 | 0.01 | 84.38 | + |
| MD23 | ENSMODG00000016780 | ENSMODP00000020944 | 1 | + | 739086525 | 739087283 | ENSMODG00000000644 | ENSMODP00000000769 | 2 | 0.20 | 0.31 | - |
| MD24 | ENSMODG00000024678 | ENSMODP00000035121 | 1 | + | 131828612 | 131829121 | ENSMODG00000003686 | ENSMODP00000004515 | 6 | 0.03 | 13.23 | - |
| MD25 | ENSMODG00000024628 | ENSMODP00000034930 | 1 | + | 146926036 | 146926614 | ENSMODG00000000403 | ENSMODP00000000482 | 2 | 0.05 | 2.78 | - |
| MD26 | ENSMODG00000024444 | ENSMODP00000034162 | 1 | + | 172326549 | 172327226 | ENSMODG00000014366 | ENSMODP00000017955 | 2 | 0.02 | 22.76 | + |
| MD27 | ENSMODG00000023945 | ENSMODP00000005916 | 1 | + | 267216513 | 267218417 | ENSMODG00000012958 | ENSMODP00000016211 | 4 | 0.01 | 12.18 | + |
| MD28 | ENSMODG00000013132 | ENSMODP00000016433 | 1 | + | 318601923 | 318602708 | ENSMODG00000014470 | ENSMODP00000032091 | 1 | 0.15 | 4.34 | + |
| MD29 | ENSMODG00000006398 | ENSMODP00000007934 | 1 | + | 371625750 | 371627015 | ENSMODG00000009574 | ENSMODP00000011964 | 1 | 0.24 | 0.14 | - |
| MD30 | ENSMODG00000023666 | ENSMODP00000031065 | 1 | + | 383246835 | 383247245 | ENSMODG00000005299 | ENSMODP00000006533 | 2 | 0.01 | 63.97 | + |
| MD31 | ENSMODG00000003673 | ENSMODP00000004498 | 1 | + | 385782151 | 385782558 | ENSMODG00000009638 | ENSMODP00000012042 | 8 | 0.01 | 59.16 | + |
| MD32 | ENSMODG00000025042 | ENSMODP00000036656 | 1 | + | 66584072 | 66585190 | ENSMODG00000002155 | ENSMODP00000002617 | 5 | 0.03 | 19.05 | + |
| MD33 | ENSMODG00000024973 | ENSMODP00000036424 | 1 | + | 72990506 | 72991792 | ENSMODG00000011590 | ENSMODP00000014502 | 4 | 0.21 | 0.30 | - |
| MD34 | ENSMODG00000012036 | ENSMODP00000015077 | 1 | + | 559295944 | 559297068 | ENSMODG00000001015 | ENSMODP00000001208 | 6 | 0.06 | 0.23 | - |
| MD35 | ENSMODG00000008634 | ENSMODP00000010741 | 1 | - | 87760616 | 87762142 | ENSMODG00000003000 | ENSMODP00000003645 | 2 | 0.34 | 0.73 | - |
| MD36 | ENSMODG00000005722 | ENSMODP00000028777 | 1 | - | 567736683 | 567738449 | ENSMODG00000007642 | ENSMODP00000009488 | 2 | 0.00 | 0.02 | - |
| MD37 | - | - | 1 | - | 693176091 | 693176666 | ENSMODG00000009451 | ENSMODP00000011806 | 8 | 0.19 | 0.28 | - |
| MD38 | ENSMODG00000023069 | ENSMODP00000028154 | 1 | - | 705389317 | 705390099 | ENSMODG00000025484 | ENSMODP00000038266 | 7 | 0.19 | 0.19 | - |
| MD39 | ENSMODG00000011125 | ENSMODP00000013924 | 1 | - | 108745303 | 108745947 | ENSMODG00000009818 | ENSMODP00000012284 | 8 | 0.01 | 31.48 | + |
| MD40 | ENSMODG00000009018 | ENSMODP00000011242 | 1 | - | 24240352 | 24241614 | ENSMODG00000013909 | ENSMODP00000017398 | 1 | 0.05 | 4.19 | - |
| MD41 | ENSMODG00000023959 | ENSMODP00000032392 | 1 | - | 262980649 | 262981719 | ENSMODG00000002224 | ENSMODP00000002709 | 4 | 0.01 | 72.10 | + |
| MD42 | - | - | 1 | - | 33722188 | 33723417 | ENSMODG00000002647 | ENSMODP00000003213 | 2 | 0.01 | 1.25 | - |
| MD43 | - | - | 1 | - | 39407253 | 39408629 | ENSMODG00000025630 | ENSMODP00000038441 | 3 | 0.18 | 1.86 | - |
| MD44 | ENSMODG00000010051 | ENSMODP00000012593 | 1 | - | 341495672 | 341496193 | ENSMODG00000000572 | ENSMODP00000000687 | 8 | 0.01 | 80.66 | - |
| MD45 | ENSMODG00000015169 | ENSMODP00000018953 | 1 | - | 524006026 | 524007099 | ENSMODG00000007697 | ENSMODP00000009558 | 6 | 0.04 | 12.23 | + |
| MD46 | ENSMODG00000016206 | ENSMODP00000020254 | 1 | - | 500417535 | 500418665 | ENSMODG00000018283 | ENSMODP00000022798 | 8 | 0.30 | 0.30 | - |
| MD47 | ENSMODG00000012963 | ENSMODP00000016217 | 1 | - | 436904584 | 436905432 | ENSMODG00000021275 | ENSMODP00000026594 | 7 | 0.01 | 20.05 | + |
| MD48 | - | - | 1 | - | 420276503 | 420277180 | ENSMODG00000006342 | ENSMODP00000007870 | 1 | 0.17 | 0.60 | - |
| MD49 | - | - | 1 | - | 418538654 | 418539664 | ENSMODG00000006522 | ENSMODP00000008092 | 5 | 0.00 | 0.01 | - |
| MD50 | - | - | 1 | - | 402249552 | 402251078 | ENSMODG00000004228 | ENSMODP00000005211 | 2 | 0.01 | 63.47 | + |
| MD51 | ENSMODG00000003709 | ENSMODP00000004542 | 2 | + | 36508021 | 36508701 | ENSMODG00000000169 | ENSMODP00000000195 | 4 | 0.22 | 0.21 | - |
| MD52 | ENSMODG00000003318 | ENSMODP00000004056 | 2 | + | 33480090 | 33481052 | ENSMODG00000001077 | ENSMODP00000001281 | 1 | 0.01 | 53.52 | + |
| MD53 | ENSMODG00000010963 | ENSMODP00000013720 | 2 | + | 207312366 | 207314396 | ENSMODG00000004756 | ENSMODP00000005860 | 3 | 0.01 | 49.94 | + |
| MD54 | ENSMODG00000013923 | ENSMODP00000017414 | 2 | + | 195972377 | 195972820 | ENSMODG00000014024 | ENSMODP00000017274 | 2 | 0.05 | 2.56 | + |
| MD55 | ENSMODG00000024606 | ENSMODP00000034856 | 2 | + | 156554426 | 156556108 | ENSMODG00000002756 | ENSMODP00000003357 | 1 | 0.18 | 0.21 | - |
| MD56 | ENSMODG00000025430 | ENSMODP00000038162 | 2 | + | 1755699 | 1756745 | ENSMODG00000007717 | ENSMODP00000009585 | 8 | 0.01 | 62.27 | + |
| MD57 | ENSMODG00000003697 | ENSMODP00000013667 | 2 | + | 36594342 | 36595571 | ENSMODG00000002190 | ENSMODP00000002662 | 2 | 0.17 | 0.23 | - |
| MD58 | ENSMODG00000016136 | ENSMODP00000020156 | 2 | + | 265517569 | 265518162 | ENSMODG00000013790 | ENSMODP00000017243 | 6 | 0.16 | 0.10 | - |
| MD59 | - | - | 2 | + | 273515550 | 273516089 | ENSMODG00000018002 | ENSMODP00000022451 | 8 | 0.06 | 11.48 | + |
| MD60 | ENSMODG00000013715 | ENSMODP00000017148 | 2 | + | 275067284 | 275069293 | ENSMODG00000012958 | ENSMODP00000016211 | 4 | 0.06 | 0.01 | - |
| MD61 | ENSMODG00000023290 | ENSMODP00000029339 | 2 | + | 422443715 | 422444548 | ENSMODG00000001775 | ENSMODP00000002171 | 6 | 0.12 | 6.52 | + |
| MD62 | ENSMODG00000023244 | ENSMODP00000029108 | 2 | + | 471582710 | 471583078 | ENSMODG00000019554 | ENSMODP00000024390 | 1 | 0.21 | 0.40 | - |
| MD63 | ENSMODG00000008521 | ENSMODP00000028589 | 2 | + | 522264100 | 522266499 | ENSMODG00000010345 | ENSMODP00000012963 | 7 | 0.21 | 0.30 | - |
| MD64 | ENSMODG00000002899 | ENSMODP00000028395 | 2 | + | 538361001 | 538363424 | ENSMODG00000008247 | ENSMODP00000010246 | 2 | 0.11 | 1.19 | + |
| MD65 | ENSMODG00000023089 | ENSMODP00000028296 | 2 | - | 540387613 | 540388464 | ENSMODG00000004890 | ENSMODP00000033592 | 4 | 0.01 | 63.63 | + |
| MD66 | - | - | 2 | - | 522928287 | 522929786 | ENSMODG00000016600 | ENSMODP00000020719 | 6 | 0.08 | 4.00 | - |
| MD67 | ENSMODG00000012840 | ENSMODP00000016067 | 2 | - | 101647857 | 101650499 | ENSMODG00000018374 | ENSMODP00000022912 | 4 | 0.06 | 8.43 | + |
| MD68 | ENSMODG00000000466 | ENSMODP00000000553 | 2 | - | 105581235 | 105582161 | ENSMODG00000013392 | ENSMODP00000016742 | 6 | 0.17 | 2.25 | + |
| MD69 | ENSMODG00000000505 | ENSMODP00000000604 | 2 | - | 105725352 | 105726782 | ENSMODG00000015379 | ENSMODP00000034444 | 8 | 0.01 | 69.00 | + |
| MD70 | ENSMODG00000011859 | ENSMODP00000014858 | 2 | - | 12296206 | 12297795 | ENSMODG00000013219 | ENSMODP00000016539 | 1 | 0.16 | 1.65 | + |
| MD71 | ENSMODG00000024474 | ENSMODP00000034272 | 2 | - | 181625744 | 181626445 | ENSMODG00000004277 | ENSMODP00000005268 | 8 | 0.34 | 0.58 | - |
| MD72 | ENSMODG00000011360 | ENSMODP00000014206 | 2 | - | 204978139 | 204979326 | ENSMODG00000013779 | ENSMODP00000027822 | 6 | 0.00 | 75.47 | + |
| MD73 | - | - | 2 | - | 36425518 | 36426348 | ENSMODG00000019055 | ENSMODP00000023789 | 5 | 0.27 | 0.28 | - |
| MD74 | - | - | 2 | - | 269413840 | 269415546 | ENSMODG00000018382 | ENSMODP00000022921 | 2 | 0.29 | 0.21 | - |
| MD75 | ENSMODG00000023171 | ENSMODP00000028722 | 2 | - | 516078305 | 516078565 | ENSMODG00000000393 | ENSMODP00000000472 | 4 | 0.02 | 15.45 | + |
| MD76 | ENSMODG00000023174 | ENSMODP00000028731 | 2 | - | 515113268 | 515113723 | ENSMODG00000009222 | ENSMODP00000011511 | 4 | 0.00 | 14.71 | - |
| MD77 | ENSMODG00000024906 | ENSMODP00000036158 | 2 | - | 81134274 | 81134558 | ENSMODG00000007222 | ENSMODP00000008950 | 2 | 0.04 | 0.26 | - |
| MD78 | ENSMODG00000017284 | ENSMODP00000021563 | 2 | - | 418390893 | 418392068 | ENSMODG00000015505 | ENSMODP00000019354 | 1 | 0.01 | 28.07 | - |
| MD79 | ENSMODG00000005764 | ENSMODP00000029543 | 2 | - | 384796131 | 384797111 | ENSMODG00000011691 | ENSMODP00000014645 | 4 | 0.13 | 0.73 | - |
| MD80 | ENSMODG00000018930 | ENSMODP00000023619 | 2 | - | 289247086 | 289248048 | ENSMODG00000012166 | ENSMODP00000015238 | 2 | 0.02 | 36.96 | + |
| MD81 | - | - | 3 | - | 56444565 | 56446481 | ENSMODG00000006222 | ENSMODP00000007717 | 3 | 0.00 | 29.18 | - |
| MD82 | ENSMODG00000024995 | ENSMODP00000036517 | 3 | + | 154411287 | 154412462 | ENSMODG00000014608 | ENSMODP00000018252 | 2 | 0.10 | 5.44 | - |
| MD83 | ENSMODG00000024788 | ENSMODP00000035629 | 3 | + | 252069566 | 252070000 | ENSMODG00000007988 | ENSMODP00000009945 | 2 | 0.07 | 0.77 | - |
| MD84 | ENSMODG00000021403 | ENSMODP00000026739 | 3 | + | 291507349 | 291508653 | ENSMODG00000011798 | ENSMODP00000014780 | 4 | 0.19 | 0.47 | - |
| MD85 | ENSMODG00000025347 | ENSMODP00000037783 | 3 | + | 33781795 | 33784545 | ENSMODG00000007267 | ENSMODP00000009005 | 8 | 0.16 | 0.48 | - |
| MD86 | - | - | 3 | + | 397262047 | 397264323 | ENSMODG00000002035 | ENSMODP00000034201 | 3 | 0.06 | 7.95 | + |
| MD87 | ENSMODG00000001681 | ENSMODP00000002057 | 3 | + | 46019814 | 46021469 | ENSMODG00000017797 | ENSMODP00000033204 | 1 | 0.01 | 68.58 | + |
| MD88 | - | - | 3 | - | 458710552 | 458711805 | ENSMODG00000002647 | ENSMODP00000003213 | 2 | 0.02 | 30.47 | + |
| MD89 | ENSMODG00000024050 | ENSMODP00000032738 | 3 | - | 439221540 | 439222064 | ENSMODG00000009580 | ENSMODP00000011969 | 5 | 0.01 | 62.01 | - |
| MD90 | ENSMODG00000010616 | ENSMODP00000013297 | 3 | - | 429144265 | 429145317 | ENSMODG00000005918 | ENSMODP00000007321 | 1 | 0.18 | 0.84 | - |
| MD91 | ENSMODG00000025020 | ENSMODP00000036603 | 3 | - | 144717560 | 144719449 | ENSMODG00000008490 | ENSMODP00000010555 | 5 | 0.32 | 0.11 | - |
| MD92 | ENSMODG00000024938 | ENSMODP00000036311 | 3 | - | 178587960 | 178588310 | ENSMODG00000014718 | ENSMODP00000018398 | 2 | 0.01 | 0.42 | - |
| MD93 | - | - | 3 | - | 195014240 | 195014776 | ENSMODG00000014484 | ENSMODP00000018102 | 1 | 0.00 | 0.02 | - |
| MD94 | ENSMODG00000025373 | ENSMODP00000037929 | 3 | - | 20311289 | 20312446 | ENSMODG00000001015 | ENSMODP00000001208 | 6 | 0.02 | 2.23 | + |
| MD95 | ENSMODG00000024783 | ENSMODP00000035614 | 3 | - | 256169126 | 256170130 | ENSMODG00000011967 | ENSMODP00000014996 | 1 | 0.29 | 0.48 | - |
| MD96 | ENSMODG00000012320 | ENSMODP00000015433 | 3 | - | 303521492 | 303522427 | ENSMODG00000015136 | ENSMODP00000018917 | 2 | 0.02 | 59.25 | + |
| MD97 | ENSMODG00000004037 | ENSMODP00000004965 | 3 | - | 392299944 | 392301239 | ENSMODG00000003104 | ENSMODP00000003792 | 7 | 0.12 | 0.71 | - |
| MD98 | - | - | 4 | + | 256218569 | 256219522 | ENSMODG00000017334 | ENSMODP00000021634 | 7 | 0.23 | 0.14 | - |
| MD99 | - | - | 4 | + | 259840990 | 259842576 | ENSMODG00000019582 | ENSMODP00000024431 | 3 | 0.06 | 0.01 | - |
| MD100 | ENSMODG00000024056 | ENSMODP00000032757 | 4 | + | 335446635 | 335447303 | ENSMODG00000023181 | ENSMODP00000007982 | 2 | 0.01 | 69.29 | + |
| MD101 | - | - | 4 | + | 346047698 | 346048393 | ENSMODG00000025468 | ENSMODP00000038245 | 8 | 0.27 | 0.18 | - |
| MD102 | - | - | 4 | + | 374961104 | 374961811 | ENSMODG00000002593 | ENSMODP00000003148 | 8 | 0.28 | 0.41 | - |
| MD103 | - | - | 4 | + | 379781893 | 379783023 | ENSMODG00000002647 | ENSMODP00000003213 | 2 | 0.07 | 3.80 | + |
| MD104 | - | - | 4 | + | 398923867 | 398924592 | ENSMODG00000015970 | ENSMODP00000019930 | 7 | 0.05 | 5.05 | - |
| MD105 | - | - | 4 | + | 411232035 | 411232685 | ENSMODG00000003527 | ENSMODP00000004305 | 5 | 0.09 | 4.19 | + |
| MD106 | ENSMODG00000014731 | ENSMODP00000018416 | 4 | + | 248613521 | 248615131 | ENSMODG00000015931 | ENSMODP00000019884 | 7 | 0.04 | 23.63 | + |
| MD107 | ENSMODG00000010451 | ENSMODP00000013099 | 4 | + | 195865639 | 195866598 | ENSMODG00000005569 | ENSMODP00000006888 | 3 | 0.21 | 0.67 | - |
| MD108 | ENSMODG00000024936 | ENSMODP00000036304 | 4 | + | 114013033 | 114013656 | ENSMODG00000008253 | ENSMODP00000010252 | 8 | 0.28 | 0.26 | - |
| MD109 | ENSMODG00000021006 | ENSMODP00000037956 | 4 | - | 6957948 | 6958652 | ENSMODG00000006609 | ENSMODP00000008198 | 2 | 0.02 | 25.82 | + |
| MD110 | - | - | 4 | - | 92463833 | 92464177 | ENSMODG00000015787 | ENSMODP00000019693 | 2 | 0.01 | 66.07 | - |
| MD111 | ENSMODG00000000972 | ENSMODP00000001160 | 4 | - | 132802571 | 132803776 | ENSMODG00000000560 | ENSMODP00000000673 | 5 | 0.04 | 11.93 | + |
| MD112 | - | - | 4 | - | 279727053 | 279728114 | ENSMODG00000007285 | ENSMODP00000009032 | 2 | 0.01 | 65.43 | - |
| MD113 | - | - | 4 | - | 353889187 | 353889924 | ENSMODG00000014740 | ENSMODP00000018428 | 1 | 0.00 | 50.50 | + |
| MD114 | ENSMODG00000002757 | ENSMODP00000003358 | 4 | - | 374546757 | 374548811 | ENSMODG00000020200 | ENSMODP00000025267 | 5 | 0.23 | 1.04 | - |
| MD115 | - | - | 4 | - | 390205809 | 390206918 | ENSMODG00000018931 | ENSMODP00000023622 | 5 | 0.14 | 1.20 | - |
| MD116 | ENSMODG00000025182 | ENSMODP00000037296 | 4 | + | 61573200 | 61574138 | ENSMODG00000011614 | ENSMODP00000014538 | 6 | 0.12 | 0.75 | - |
| MD117 | ENSMODG00000018937 | ENSMODP00000023629 | 5 | + | 8184236 | 8184739 | ENSMODG00000007323 | ENSMODP00000009084 | 8 | 0.02 | 3.65 | - |
| MD118 | ENSMODG00000000016 | ENSMODP00000000016 | 5 | + | 144011304 | 144012227 | ENSMODG00000019001 | ENSMODP00000023713 | 5 | 0.16 | 1.12 | - |
| MD119 | ENSMODG00000000014 | ENSMODP00000000012 | 5 | + | 144324142 | 144324597 | ENSMODG00000015439 | ENSMODP00000019271 | 6 | 0.27 | 0.32 | - |
| MD120 | ENSMODG00000001155 | ENSMODP00000001376 | 5 | + | 215466943 | 215467647 | ENSMODG00000002366 | ENSMODP00000002879 | 4 | 0.01 | 71.33 | - |
| MD121 | ENSMODG00000004184 | ENSMODP00000005162 | 5 | + | 233258515 | 233259138 | ENSMODG00000007582 | ENSMODP00000009409 | 5 | 0.10 | 0.62 | - |
| MD122 | ENSMODG00000025078 | ENSMODP00000036795 | 5 | + | 81826176 | 81827042 | ENSMODG00000020593 | ENSMODP00000025757 | 5 | 0.02 | 3.79 | - |
| MD123 | ENSMODG00000025096 | ENSMODP00000036879 | 5 | + | 78322924 | 78324507 | ENSMODG00000004444 | ENSMODP00000005474 | 1 | 0.02 | 0.37 | - |
| MD124 | ENSMODG00000025135 | ENSMODP00000037142 | 5 | - | 52215998 | 52216540 | ENSMODG00000010354 | ENSMODP00000012972 | 7 | 0.02 | 6.67 | - |
| MD125 | - | - | 5 | - | 307478130 | 307479410 | ENSMODG00000018928 | ENSMODP00000030290 | 2 | 0.01 | 27.11 | + |
| MD126 | ENSMODG00000009912 | ENSMODP00000012405 | 5 | - | 270118233 | 270119114 | ENSMODG00000001559 | ENSMODP00000001904 | 8 | 0.01 | 56.70 | + |
| MD127 | ENSMODG00000000474 | ENSMODP00000000562 | 5 | - | 203698734 | 203699447 | ENSMODG00000021041 | ENSMODP00000026310 | 4 | 0.01 | 64.31 | + |
| MD128 | ENSMODG00000024812 | ENSMODP00000035731 | 5 | - | 184072454 | 184073026 | ENSMODG00000007914 | ENSMODP00000009847 | 6 | 0.07 | 7.21 | + |
| MD129 | - | - | 5 | - | 107831515 | 107832909 | ENSMODG00000015977 | ENSMODP00000019944 | 2 | 0.01 | 38.01 | + |
| MD130 | ENSMODG00000025196 | ENSMODP00000037350 | 6 | + | 31302337 | 31303887 | ENSMODG00000012189 | ENSMODP00000015261 | 3 | 0.07 | 2.68 | + |
| MD131 | ENSMODG00000025211 | ENSMODP00000037373 | 6 | + | 29501154 | 29502155 | ENSMODG00000018066 | ENSMODP00000022521 | 8 | 0.13 | 0.61 | - |
| MD132 | - | - | 6 | + | 239407308 | 239407895 | ENSMODG00000006616 | ENSMODP00000008205 | 2 | 0.16 | 0.82 | - |
| MD133 | ENSMODG00000003631 | ENSMODP00000004445 | 6 | + | 102078916 | 102080310 | ENSMODG00000004854 | ENSMODP00000005976 | 1 | 0.02 | 44.89 | + |
| MD134 | - | - | 6 | - | 205995402 | 205996103 | ENSMODG00000005254 | ENSMODP00000006478 | 8 | 0.01 | 21.45 | + |
| MD135 | ENSMODG00000025351 | ENSMODP00000037808 | 6 | - | 13957716 | 13958210 | ENSMODG00000016089 | ENSMODP00000020088 | 1 | 0.27 | 0.41 | - |
| MD136 | ENSMODG00000024840 | ENSMODP00000035827 | 6 | - | 100183912 | 100184727 | ENSMODG00000002317 | ENSMODP00000002824 | 1 | 0.19 | 2.50 | - |
| MD137 | - | - | 7 | + | 201092003 | 201092941 | ENSMODG00000011517 | ENSMODP00000014401 | 1 | 0.00 | 71.38 | - |
| MD138 | - | - | 7 | + | 191926075 | 191926866 | ENSMODG00000013151 | ENSMODP00000016457 | 4 | 0.02 | 0.97 | - |
| MD139 | - | - | 7 | + | 189029908 | 189031605 | ENSMODG00000006737 | ENSMODP00000033322 | 8 | 0.00 | 59.13 | + |
| MD140 | - | - | 7 | + | 75769909 | 75770340 | ENSMODG00000010312 | ENSMODP00000012927 | 3 | 0.01 | 60.55 | + |
| MD141 | ENSMODG00000003192 | ENSMODP00000003887 | 7 | + | 75769048 | 75769458 | ENSMODG00000010312 | ENSMODP00000012927 | 3 | 0.00 | 74.22 | + |
| MD142 | - | - | 7 | + | 75767191 | 75767634 | ENSMODG00000010312 | ENSMODP00000012927 | 3 | 0.08 | 4.36 | + |
| MD143 | - | - | 7 | - | 242576612 | 242577319 | ENSMODG00000004277 | ENSMODP00000005268 | 8 | 0.32 | 0.83 | - |
| MD144 | - | - | 7 | - | 215682986 | 215683888 | ENSMODG00000013383 | ENSMODP00000016734 | 6 | 0.29 | 0.46 | - |
| MD145 | - | - | 7 | - | 211945993 | 211947090 | ENSMODG00000015696 | ENSMODP00000019589 | 6 | 0.26 | 0.96 | - |
| MD146 | - | - | 7 | - | 191439559 | 191441097 | ENSMODG00000011257 | ENSMODP00000014090 | 6 | 0.01 | 64.68 | - |
| MD147 | - | - | 8 | + | 122327609 | 122331289 | ENSMODG00000007253 | ENSMODP00000008992 | 5 | 0.01 | 40.37 | - |
| MD148 | - | - | 8 | + | 205286063 | 205286827 | ENSMODG00000001586 | ENSMODP00000001936 | 7 | 0.11 | 1.36 | - |
| MD149 | - | - | 8 | + | 232164095 | 232165213 | ENSMODG00000012003 | ENSMODP00000015042 | 3 | 0.01 | 32.91 | - |
| MD150 | - | - | 8 | + | 262203047 | 262205641 | ENSMODG00000015890 | ENSMODP00000019829 | 2 | 0.15 | 0.28 | - |
| MD151 | - | - | 8 | + | 263065640 | 263066401 | ENSMODG00000004242 | ENSMODP00000005228 | 1 | 0.21 | 0.47 | - |
| MD152 | - | - | 8 | + | 289572498 | 289573088 | ENSMODG00000016496 | ENSMODP00000020596 | Un | 0.08 | 1.34 | - |
| MD153 | - | - | 8 | + | 307365548 | 307366120 | ENSMODG00000016078 | ENSMODP00000032375 | 4 | 0.03 | 2.97 | - |
| MD154 | - | - | 8 | + | 119244753 | 119245229 | ENSMODG00000009222 | ENSMODP00000011511 | 4 | 0.00 | 25.67 | - |
| MD155 | - | - | 8 | + | 117551658 | 117552119 | ENSMODG00000009222 | ENSMODP00000011511 | 4 | 0.00 | 43.51 | + |
| MD156 | - | - | 8 | + | 79636830 | 79637438 | ENSMODG00000007134 | ENSMODP00000008849 | 2 | 0.04 | 29.90 | - |
| MD157 | - | - | 8 | + | 11648527 | 11648730 | ENSMODG00000003890 | ENSMODP00000004765 | 3 | 0.16 | 0.28 | - |
| MD158 | - | - | 8 | - | 294413994 | 294414359 | ENSMODG00000020362 | ENSMODP00000025467 | 5 | 0.17 | 0.93 | - |
| MD159 | ENSMODG00000010440 | ENSMODP00000013088 | 8 | - | 262649912 | 262650937 | ENSMODG00000016902 | ENSMODP00000021091 | 8 | 0.25 | 0.25 | - |
| MD160 | - | - | 8 | - | 166074661 | 166075521 | ENSMODG00000005921 | ENSMODP00000007331 | 3 | 0.20 | 1.77 | - |
| MD161 | - | - | 8 | - | 95750037 | 95750936 | ENSMODG00000015812 | ENSMODP00000019731 | 8 | 0.03 | 17.97 | + |
| MD162 | ENSMODG00000007284 | ENSMODP00000009030 | 8 | - | 10709571 | 10710308 | ENSMODG00000021103 | ENSMODP00000026384 | 4 | 0.01 | 57.73 | + |
| MD163 | - | - | 8 | - | 61358331 | 61360044 | ENSMODG00000012753 | ENSMODP00000015957 | 6 | 0.03 | 11.87 | + |
| MD164 | - | - | 8 | + | 307661849 | 307662559 | ENSMODG00000017505 | ENSMODP00000021838 | 7 | 0.12 | 0.43 | - |

^­­^

^1^ ENSG and ENSP are Ensemble identifier for genes and proteins, respectively.

^2^ “Human shared” indicates whether our dating procedure detects an orthologous retrogene in the human genome. ‘und’ stands for undefined.
