# Supplementary material for: Chromosomal Gene Movements Reflect the Recent Origin and Biology of Therian Sex Chromosomes
Source: PLoS Biol. 2008 Apr 1;6(4):e80. doi: 10.1371/journal.pbio.0060080 (PMC2276528; doi:10.1371/journal.pbio.0060080)
Supplement: Supplementary file 6 [file pbio.0060080.st005.doc]

**Table S5.** Mouse parental gene and retrogene expression data.

| Retro. ID | Parent name^1^ | Par. chr. | Par TS^2^ | Par. somatic expr.^3^ | Par. SG^4^ | Par. SC^4^ | Par. ST^4^ | Par. TT^4^ | Par. stage^5^ | Retrogene name^1^ | Retro. chr. | Retro. TS^2^ | Retro. somatic expr.^3^ | Retro. SG^4^ | Retro. SC^4^ | Retro. ST^4^ | Retro. TT^4^ | Retro. stage^5^ |
| --- | --- | --- | --- | --- | --- | --- | --- | --- | --- | --- | --- | --- | --- | --- | --- | --- | --- | --- |
| MM1 | *Ddx3x* | X | - | 14 | 11.40 | 5.10 | 4.06 | 7.10 | MI | *D1Pas1* | 1 | + | 0 | 6.21 | 11.00 | 10.30 | 10.40 | ME |
| MM2 | *Prps1* | X | - | 14 | 8.56 | 5.32 | 6.85 | 6.90 | MI | *1700011K15Rik* | 12 | + | 0 | 4.15 | 12.00 | 11.45 | 11.60 | ME |
| MM3 | *Phf8* | X | - | 14 | 8.35 | 5.01 | 5.90 | 6.50 | SO | *NP_001009544.1* | 17 | + | 0 | 4.20 | 6.43 | 9.10 | 8.06 | PM |
| MM4 | *Nxt2* | X | - | 9 | 5.71 | 3.11 | 4.10 | 3.95 | SO | *Nxt1* | 2 | - | 14 | 9.36 | 12.10 | 11.00 | 11.25 | ME |
| MM5 | *2400002F11Rik* | X | - | 14 | 8.51 | 4.80 | 4.45 | 5.96 | SO | *-* | 2 | - | 14 | 8.60 | 9.70 | 10.05 | 10.00 | - |
| MM6 | *Pgk1* | X | - | 14 | 10.66 | 6.45 | 7.35 | 8.35 | SO | *Pgk2* | 3 | + | 0 | 3.46 | 11.05 | 12.40 | 12.35 | ME |
| MM7 | *UBL4_MOUSE* | X | - | 14 | 9.15 | 6.26 | 7.95 | 7.40 | SO | *-* | 3 | + | 0 | 3.96 | 8.35 | 11.15 | 11.50 | PM |
| MM8 | *Pdha1* | X | - | 14 | 10.20 | 5.51 | 5.43 | 6.70 | SO | *Pdha2* | 3 | + | 0 | 6.15 | 11.85 | 11.05 | 11.50 | ME |
| MM9 | *Gyk* | X | - | 8 | 3.67 | 2.95 | 3.10 | 3.20 | - | *Gk-rs1* | 5 | + | 0 | 3.05 | 13.05 | 12.95 | 12.70 | ME |
| MM10 | *Igbp1* | X | - | 14 | 9.67 | 5.61 | 5.76 | 6.86 | MI | *Igbp1b* | 6 | + | 0 | 4.45 | 9.05 | 9.55 | 9.20 | ME |
| MM11 | *Rnf128* | X | - | 10 | 6.31 | 4.40 | 4.60 | 5.65 | SO | *Rnf133* | 6 | + | 0 | 3.75 | 8.20 | 10.96 | 11.05 | PM |
| MM12 | *Rps4x* | X | - | 14 | 13.25 | 6.06 | 6.00 | 9.00 | MI | *-* | 6 | - | 14 | 11.71 | 11.05 | 10.95 | 10.95 | - |
| MM13 | *Rbmx* | X | - | 14 | 7.17 | 4.66 | 5.30 | 4.95 | - | *-* | 7 | - | 3 | 7.92 | 10.00 | 10.20 | 10.05 | ME |
| MM14 | *Idh3g* | X | - | 14 | 10.35 | 5.30 | 6.60 | 7.80 | MI | *-* | 7 | + | 0 | 4.50 | 7.91 | 10.35 | 9.70 | PM |
| MM15 | *U2af1-rs2* | X | N.D.. | N.D. | N.D. | N.D. | N.D. | N.D. | N.D. | *Commd1* | 11 | - | 14 | 7.93 | 7.20 | 7.26 | 7.35 | - |
| MM16 | *XAP5_MOUSE* | X | N.D. | N.D. | N.D. | N.D. | N.D. | N.D. | N.D. | *XAP5L_MOUSE* | 13 | + | 0 | 4.39 | 10.90 | 10.70 | 10.75 | ME |
| MM17 | *Cetn2* | X | N.D. | N.D. | N.D. | N.D. | N.D. | N.D. | N.D. | *Cetn1* | 18 | + | 0 | 3.50 | 12.25 | 12.05 | 11.95 | ME |
| MM18 | *Utp14a* | X | - | 14 | 8.40 | 6.36 | 8.10 | 7.60 | - | *Utp14b* | 1 | N.D. | N.D. | N.D. | N.D. | N.D. | N.D. | N.D. |
| MM19 | *C6.1A* | X | - | 14 | 8.16 | 5.53 | 5.39 | 5.90 | MI | *-* | 10 | N.D. | N.D. | N.D. | N.D. | N.D. | N.D. | N.D. |
| MM20 | *Kif4* | X | - | 3 | 7.56 | 4.80 | 5.35 | 5.00 | MI | *XP_138220.2* | 12 | N.D. | N.D. | N.D. | N.D. | N.D. | N.D. | N.D. |
| MM21 | *Plp2* | X | - | 14 | 10.66 | 5.23 | 5.49 | 6.01 | SO | *-* | 13 | N.D. | N.D. | N.D. | N.D. | N.D. | N.D. | N.D. |
| MM22 | *Fundc2* | X | - | 14 | 9.30 | 4.66 | 4.97 | 5.90 | SO | *1700034I23Rik* | 3 | N.D. | N.D. | N.D. | N.D. | N.D. | N.D. | N.D. |
| MM23 | *Klhl13* | X | - | 10 | 8.47 | 3.35 | 3.30 | 3.41 | - | *-* | 4 | N.D. | N.D. | N.D. | N.D. | N.D. | N.D. | N.D. |
| MM24 | *-* | X | - | 10 | 8.20 | 5.10 | 6.30 | 6.01 | SO | *-* | 5 | N.D. | N.D. | N.D. | N.D. | N.D. | N.D. | N.D. |
| MM25 | *Dnajb6* | 5 | - | 7 | 6.55 | 11.40 | 11.40 | 11.20 | ME | *Dnajb3* | 1 | + | 0 | 5.66 | 12.25 | 12.80 | 12.75 | ME |
| MM26 | *Zfp148* | 16 | - | 14 | 9.25 | 7.10 | 6.36 | 7.35 | MI | *Zfp281* | 1 | - | 14 | 10.00 | 8.75 | 6.25 | 8.15 | MI |
| MM27 | *Cs* | 10 | - | 14 | 11.05 | 9.15 | 7.90 | 9.10 | MI | *Csl* | 10 | + | 0 | 4.51 | 10.65 | 10.60 | 10.50 | - |
| MM28 | *2410003A14Rik* | 7 | - | 14 | 10.00 | 9.00 | 6.50 | 8.55 | MI | *-* | 10 | + | 0 | 4.00 | 12.60 | 12.20 | 12.05 | ME |
| MM29 | *Stard5* | 7 | - | 14 | 6.70 | 5.56 | 5.66 | 5.80 | - | *Stard6* | 10 | - | 12 | 4.80 | 12.05 | 12.55 | 12.70 | ME |
| MM30 | *Phb* | 11 | - | 14 | 10.90 | 6.91 | 8.10 | 8.65 | MI | *-* | 11 | - | 2 | 5.60 | 5.85 | 6.50 | 6.35 | - |
| MM31 | *Uba52* | 8 | - | 14 | 14.15 | 13.15 | 13.65 | 13.85 | - | *-* | 11 | - | 14 | 6.75 | 5.76 | 6.80 | 6.35 | - |
| MM32 | *Aldoa* | 7 | - | 14 | 11.75 | 10.20 | 11.60 | 11.75 | - | *-* | 12 | + | 0 | 4.85 | 9.06 | 11.80 | 11.40 | PM |
| MM33 | *Arf3* | 15 | - | 14 | 10.60 | 9.30 | 8.80 | 9.25 | - | *Arf6* | 12 | - | 14 | 10.60 | 9.35 | 8.65 | 8.81 | - |
| MM34 | *Pfn1* | 11 | - | 14 | 11.95 | 10.95 | 8.95 | 10.46 | MI | *Pfn3* | 13 | + | 0 | 5.86 | 8.60 | 10.85 | 10.80 | PM |
| MM35 | *A930025D01Rik* | 2 | - | 10 | 6.70 | 8.65 | 7.50 | 7.70 | - | *NP_758497.1* | 15 | - | 14 | 7.00 | 5.51 | 6.26 | 5.95 | - |
| MM36 | *Wdr5* | 2 | - | 14 | 9.70 | 8.40 | 7.60 | 8.25 | - | *Wdr5b* | 16 | - | 1 | 4.36 | 5.10 | 4.45 | 4.51 | - |
| MM37 | *Atp6v1e1* | 6 | - | 14 | 10.10 | 9.65 | 7.75 | 9.30 | SO | *Atp6v1e2* | 17 | - | 1 | 5.27 | 9.41 | 11.75 | 11.45 | PM |
| MM38 | *Ldh3* | 7 | - | 3 | 4.53 | 14.00 | 13.80 | 13.85 | ME | *-* | 17 | + | 0 | 4.09 | 13.05 | 12.35 | 12.50 | ME |
| MM39 | *Rnf13* | 3 | - | 14 | 8.80 | 7.20 | 6.20 | 7.15 | SO | *Znrf4* | 17 | - | 0 | 5.15 | 10.10 | 12.80 | 12.65 | PM |
| MM40 | *Txn1* | 4 | - | 14 | 11.70 | 5.85 | 5.79 | 8.40 | MI | *-* | 17 | - | 2 | 4.69 | 9.40 | 11.85 | 12.15 | PM |
| MM41 | *1810054D07Rik* | 4 | + | 0 | 5.26 | 7.15 | 6.50 | 6.60 | - | *4930560E09Rik* | 17 | + | 0 | 4.40 | 9.50 | 10.55 | 10.20 | ME |
| MM42 | *Actb* | 11 | - | 14 | 13.20 | 13.35 | 12.30 | 12.80 | - | *-* | 17 | + | 0 | 4.70 | 10.65 | 11.85 | 12.10 | PM |
| MM43 | *Slc25a15* | 8 | - | 14 | 7.46 | 3.75 | 3.65 | 3.70 | MI | *Slc25a2* | 18 | + | 0 | 4.39 | 9.20 | 9.90 | 9.25 | ME |
| MM44 | *Pabpc1* | 15 | - | 14 | 12.95 | 13.90 | 13.50 | 13.55 | - | *4932702K14Rik* | 18 | + | 0 | 4.10 | 12.90 | 12.80 | 12.45 | ME |
| MM45 | *Fth1* | 19 | - | 14 | 13.20 | 12.15 | 10.95 | 12.25 | - | *Ftmt* | 18 | + | 0 | 3.96 | 8.51 | 11.10 | 10.15 | PM |
| MM46 | *Ranbp5* | 14 | - | 14 | 10.15 | 12.10 | 12.90 | 12.85 | PM | *Ranbp6* | 19 | - | 6 | 5.90 | 6.85 | 5.95 | 6.40 | - |
| MM47 | *Sc4mol* | 8 | - | 13 | 9.96 | 4.95 | 5.20 | 7.30 | SO | *Ch25h* | 19 | - | 2 | 4.16 | 4.30 | 4.35 | 3.95 | - |
| MM48 | *BAT5_MOUSE* | 17 | - | 14 | 8.85 | 10.40 | 9.90 | 9.90 | - | *NP_899004.1* | 2 | + | 0 | 4.01 | 5.50 | 7.90 | 8.15 | PM |
| MM49 | *BC051244* | 6 | - | 12 | 6.41 | 5.90 | 5.70 | 6.05 | - | *Thnsl1* | 2 | - | 8 | 6.73 | 9.15 | 10.00 | 9.50 | ME |
| MM50 | *Dnajb6* | 5 | - | 7 | 6.55 | 11.40 | 11.40 | 11.20 | ME | *Dnajb3* | 3 | + | 0 | 3.37 | 8.55 | 11.45 | 11.65 | PM |
| MM51 | *Acta1* | 8 | - | 7 | 4.26 | 4.25 | 4.65 | 4.20 | - | *Arpm1* | 3 | + | 0 | 4.05 | 7.10 | 9.50 | 10.06 | PM |
| MM52 | *Tram1* | 1 | - | 14 | 10.10 | 10.85 | 9.55 | 9.95 | - | *Tram1l1* | 3 | - | 3 | 5.21 | 3.70 | 3.81 | 3.65 | - |
| MM53 | *Oxct1* | 15 | - | 14 | 10.75 | 9.25 | 10.25 | 9.85 | - | *-* | 4 | + | 0 | 4.65 | 8.75 | 11.80 | 12.05 | PM |
| MM54 | *Ywhaz* | 15 | - | 14 | 12.40 | 11.35 | 10.15 | 10.85 | - | *-* | 4 | - | 3 | 6.15 | 5.70 | 6.26 | 6.10 | - |
| MM55 | *AI553587* | 11 | - | 14 | 7.65 | 9.10 | 6.50 | 8.10 | - | *Ppp3r2* | 4 | + | 0 | 3.91 | 12.25 | 11.80 | 11.80 | ME |
| MM56 | *Actb* | 11 | - | 14 | 13.20 | 13.35 | 12.30 | 12.80 | - | *-* | 4 | - | 14 | 5.91 | 11.90 | 13.05 | 12.80 | ME |
| MM57 | *Ran* | 5 | - | 14 | 12.60 | 13.05 | 12.70 | 12.75 | - | *-* | 4 | + | 0 | 4.10 | 10.65 | 13.00 | 13.10 | PM |
| MM58 | *Aldh2* | 5 | - | 14 | 10.95 | 6.30 | 5.35 | 9.65 | MI | *Aldh1b1* | 4 | - | 3 | 7.46 | 5.20 | 5.25 | 7.15 | - |
| MM59 | *Gpd1l* | 9 | - | 14 | 7.26 | 4.15 | 4.35 | 5.56 | MI | *-* | 5 | - | 1 | 4.86 | 8.15 | 10.60 | 10.77 | PM |
| MM60 | *Cct8* | 16 | - | 14 | 11.40 | 11.05 | 9.95 | 10.50 | - | *Gm443* | 5 | - | 1 | 4.55 | 7.60 | 9.85 | 9.96 | PM |
| MM61 | *-* | 16 | - | 14 | 11.90 | 9.50 | 11.30 | 11.40 | - | *Sod3* | 5 | - | 4 | 8.10 | 5.86 | 6.46 | 7.85 | - |
| MM62 | *Rac1* | 5 | - | 14 | 12.45 | 7.40 | 6.36 | 9.30 | MI | *-* | 5 | - | 3 | 4.60 | 4.50 | 5.05 | 4.56 | - |
| MM63 | *Pcbp2* | 15 | - | 14 | 11.95 | 12.50 | 11.65 | 12.10 | - | *-* | 6 | - | 14 | 11.85 | 11.90 | 8.00 | 10.90 | MI |
| MM64 | *Nup50* | 15 | - | 14 | 9.67 | 10.75 | 11.35 | 11.00 | - | *XP_485809.1* | 6 | + | 0 | 3.71 | 13.05 | 12.95 | 12.75 | ME |
| MM65 | *1200003C05Rik* | 12 | - | 14 | 9.75 | 5.50 | 6.05 | 7.25 | MI | *4931417E11Rik* | 6 | - | 0 | 5.12 | 11.05 | 10.70 | 10.60 | ME |
| MM66 | *Capza2* | 6 | - | 14 | 10.65 | 7.60 | 6.40 | 8.15 | MI | *Capza1* | 6 | + | 0 | 3.16 | 9.25 | 11.70 | 11.75 | PM |
| MM67 | *Rhoa* | 9 | - | 14 | 11.25 | 9.15 | 8.35 | 9.00 | SO | *Rhob* | 6 | + | 0 | 3.31 | 12.20 | 12.10 | 11.85 | ME |
| MM68 | *Rac1* | 5 | - | 14 | 12.45 | 7.40 | 6.36 | 9.30 | MI | *-* | 7 | - | 14 | 8.36 | 6.10 | 6.16 | 6.40 | SO |
| MM69 | *Ubqln1* | 13 | - | 12 | 8.90 | 9.70 | 10.00 | 9.60 | - | *4931431F19Rik* | 7 | + | 0 | 3.63 | 6.85 | 9.45 | 9.77 | PM |
| MM70 | *Tesc* | 5 | - | 14 | 10.02 | 6.21 | 6.36 | 6.75 | SO | *1700008P20Rik* | 7 | + | 0 | 5.20 | 8.80 | 11.20 | 11.00 | PM |
| MM71 | *Tkt* | 14 | - | 14 | 11.50 | 7.55 | 6.46 | 8.50 | MI | *4933401I19Rik* | 8 | + | 0 | 5.53 | 9.10 | 9.05 | 8.70 | ME |
| MM72 | *Ptgis* | 2 | - | 13 | 8.95 | 6.20 | 6.51 | 7.60 | SO | *Cyp8b1* | 9 | - | 2 | 4.90 | 5.26 | 5.45 | 5.06 | - |
| MM73 | *Fbl* | 7 | N.D. | N.D. | N.D. | N.D. | N.D. | N.D. | N.D. | *NP_001004147.1* | 11 | - | 13 | 5.60 | 6.20 | 6.85 | 6.40 | - |
| MM74 | *-* | 3 | N.D. | N.D. | N.D. | N.D. | N.D. | N.D. | N.D. | *-* | 3 | - | 14 | 9.05 | 10.15 | 10.45 | 10.15 | - |
| MM75 | *Erh* | 12 | N.D. | N.D. | N.D. | N.D. | N.D. | N.D. | N.D. | *-* | 3 | + | 0 | 9.35 | 12.95 | 12.35 | 12.40 | ME |
| MM76 | *-* | 9 | N.D. | N.D. | N.D. | N.D. | N.D. | N.D. | N.D. | *Zfp29* | 7 | + | 0 | 4.71 | 7.60 | 8.40 | 7.56 | ME |
| MM77 | *-* | 2 | N.D. | N.D. | N.D. | N.D. | N.D. | N.D. | N.D. | *9030612M13Rik* | 9 | + | 0 | 3.85 | 7.05 | 8.70 | 7.60 | PM |
| MM78 | *Gle1l* | 2 | - | 14 | 8.50 | 8.00 | 8.90 | 8.46 | - | *-* | 1 | N.D. | N.D. | N.D. | N.D. | N.D. | N.D. | N.D. |
| MM79 | *Gabarapl2* | 8 | - | 14 | 10.05 | 9.00 | 9.15 | 9.40 | - | *-* | 1 | N.D. | N.D. | N.D. | N.D. | N.D. | N.D. | N.D. |
| MM80 | *Becn1* | 11 | - | 14 | 9.15 | 10.05 | 9.50 | 9.60 | - | *XP_129608.2* | 1 | N.D. | N.D. | N.D. | N.D. | N.D. | N.D. | N.D. |
| MM81 | *D17Ertd441e* | 17 | - | 11 | 9.56 | 5.10 | 4.40 | 5.56 | MI | *1700049L16Rik* | 10 | N.D. | N.D. | N.D. | N.D. | N.D. | N.D. | N.D. |
| MM82 | *Galnt12* | 4 | - | 6 | 7.66 | 4.55 | 4.60 | 5.30 | MI | *-* | 10 | N.D. | N.D. | N.D. | N.D. | N.D. | N.D. | N.D. |
| MM83 | *Hspd1* | 1 | - | 14 | 12.10 | 7.10 | 7.35 | 9.26 | MI | *-* | 11 | N.D. | N.D. | N.D. | N.D. | N.D. | N.D. | N.D. |
| MM84 | *Ube2r2* | 4 | - | 14 | 9.60 | 7.90 | 7.86 | 8.25 | - | *Cdc34* | 11 | N.D. | N.D. | N.D. | N.D. | N.D. | N.D. | N.D. |
| MM85 | *Rps16* | 7 | - | 14 | 13.85 | 12.45 | 12.00 | 12.70 | - | *-* | 11 | N.D. | N.D. | N.D. | N.D. | N.D. | N.D. | N.D. |
| MM86 | *Rps11* | 7 | - | 14 | 13.15 | 11.90 | 11.30 | 11.75 | - | *-* | 12 | N.D. | N.D. | N.D. | N.D. | N.D. | N.D. | N.D. |
| MM87 | *D9Wsu20e* | 9 | - | 14 | 9.75 | 9.90 | 9.60 | 9.90 | - | *Tmem30b* | 12 | N.D. | N.D. | N.D. | N.D. | N.D. | N.D. | N.D. |
| MM88 | *Rpl26* | 11 | - | 14 | 13.85 | 13.25 | 13.10 | 13.30 | - | *-* | 12 | N.D. | N.D. | N.D. | N.D. | N.D. | N.D. | N.D. |
| MM89 | *Calm2* | 7 | - | 14 | 8.61 | 11.90 | 11.75 | 11.70 | - | *Calml3* | 13 | N.D. | N.D. | N.D. | N.D. | N.D. | N.D. | N.D. |
| MM90 | *Actb* | 5 | - | 14 | 13.80 | 12.00 | 12.85 | 13.15 | - | *4732495G21Rik* | 13 | N.D. | N.D. | N.D. | N.D. | N.D. | N.D. | N.D. |
| MM91 | *Drd2* | 9 | - | 6 | 4.55 | 4.90 | 5.70 | 5.06 | - | *Htr1a* | 13 | N.D. | N.D. | N.D. | N.D. | N.D. | N.D. | N.D. |
| MM92 | *Cox5b* | 1 | - | 14 | 12.35 | 12.20 | 11.80 | 12.00 | - | *Cox5b* | 13 | N.D. | N.D. | N.D. | N.D. | N.D. | N.D. | N.D. |
| MM93 | *Arf5* | 6 | - | 14 | 9.20 | 8.35 | 7.66 | 8.40 | - | *Arl11* | 14 | N.D. | N.D. | N.D. | N.D. | N.D. | N.D. | N.D. |
| MM94 | *Slc5a1* | 5 | - | 4 | 4.10 | 4.25 | 4.50 | 4.20 | - | *Slc5a3* | 16 | N.D. | N.D. | N.D. | N.D. | N.D. | N.D. | N.D. |
| MM95 | *Ahcy* | 2 | - | 14 | 11.00 | 8.55 | 7.95 | 8.65 | MI | *XP_619602.1* | 16 | N.D. | N.D. | N.D. | N.D. | N.D. | N.D. | N.D. |
| MM96 | *Fgf11* | 11 | - | 5 | 5.35 | 4.66 | 4.76 | 4.40 | - | *NP_808386.1* | 16 | N.D. | N.D. | N.D. | N.D. | N.D. | N.D. | N.D. |
| MM97 | *Mrps10* | 17 | - | 14 | 9.26 | 9.55 | 7.61 | 9.10 | - | *-* | 17 | N.D. | N.D. | N.D. | N.D. | N.D. | N.D. | N.D. |
| MM98 | *2310042M24Rik* | 2 | - | 14 | 9.40 | 8.10 | 7.85 | 8.50 | - | *-* | 17 | N.D. | N.D. | N.D. | N.D. | N.D. | N.D. | N.D. |
| MM99 | *Mrpl27* | 11 | - | 14 | 10.16 | 8.85 | 6.80 | 8.70 | MI | *-* | 18 | N.D. | N.D. | N.D. | N.D. | N.D. | N.D. | N.D. |
| MM100 | *Slc35a3* | 3 | - | 10 | 5.35 | 8.75 | 9.45 | 8.65 | ME | *Slc35a4* | 18 | N.D. | N.D. | N.D. | N.D. | N.D. | N.D. | N.D. |
| MM101 | *Rpl21* | 5 | - | 2 | 5.20 | 4.40 | 4.00 | 4.16 | - | *-* | 18 | N.D. | N.D. | N.D. | N.D. | N.D. | N.D. | N.D. |
| MM102 | *Tpd52l2* | 2 | - | 14 | 9.16 | 6.25 | 6.07 | 7.00 | MI | *4931412G03Rik* | 19 | N.D. | N.D. | N.D. | N.D. | N.D. | N.D. | N.D. |
| MM103 | *Ldh2* | 6 | - | 14 | 11.55 | 7.85 | 7.70 | 10.15 | MI | *XP_484784.1* | 19 | N.D. | N.D. | N.D. | N.D. | N.D. | N.D. | N.D. |
| MM104 | *Tor2a* | 2 | - | 14 | 8.15 | 6.85 | 6.01 | 6.75 | - | *A830007P12Rik* | 2 | N.D. | N.D. | N.D. | N.D. | N.D. | N.D. | N.D. |
| MM105 | *Glo1* | 17 | - | 14 | 9.86 | 7.20 | 6.90 | 8.10 | MI | *Glo1* | 2 | N.D. | N.D. | N.D. | N.D. | N.D. | N.D. | N.D. |
| MM106 | *Pin1* | 9 | - | 14 | 10.15 | 9.95 | 7.80 | 9.40 | - | *-* | 2 | N.D. | N.D. | N.D. | N.D. | N.D. | N.D. | N.D. |
| MM107 | *Zfand1* | 3 | - | 8 | 6.08 | 3.95 | 3.80 | 3.90 | - | *-* | 2 | N.D. | N.D. | N.D. | N.D. | N.D. | N.D. | N.D. |
| MM108 | *BC056474* | 8 | - | 14 | 8.60 | 7.60 | 6.00 | 7.55 | - | *-* | 2 | N.D. | N.D. | N.D. | N.D. | N.D. | N.D. | N.D. |
| MM109 | *Pgam1* | 19 | - | 14 | 11.35 | 7.15 | 6.40 | 8.50 | MI | *-* | 3 | N.D. | N.D. | N.D. | N.D. | N.D. | N.D. | N.D. |
| MM110 | *Apg4b* | 1 | - | 14 | 8.85 | 8.75 | 8.60 | 8.75 | - | *Apg4a* | 3 | N.D. | N.D. | N.D. | N.D. | N.D. | N.D. | N.D. |
| MM111 | *Fgl1* | 8 | - | 3 | 4.87 | 4.66 | 4.60 | 4.56 | - | *XP_485259.1* | 3 | N.D. | N.D. | N.D. | N.D. | N.D. | N.D. | N.D. |
| MM112 | *Lamr1* | 9 | - | 14 | 13.35 | 10.85 | 10.55 | 11.05 | MI | *-* | 3 | N.D. | N.D. | N.D. | N.D. | N.D. | N.D. | N.D. |
| MM113 | *Ptp4a2* | 4 | - | 14 | 11.45 | 10.50 | 9.95 | 10.36 | - | *-* | 4 | N.D. | N.D. | N.D. | N.D. | N.D. | N.D. | N.D. |
| MM114 | *Got2* | 8 | - | 14 | 10.80 | 6.60 | 6.85 | 7.85 | SO | *-* | 5 | N.D. | N.D. | N.D. | N.D. | N.D. | N.D. | N.D. |
| MM115 | *4930542G03Rik* | 2 | - | 14 | 12.56 | 13.45 | 13.35 | 13.35 | - | *-* | 5 | N.D. | N.D. | N.D. | N.D. | N.D. | N.D. | N.D. |
| MM116 | *Gnpda1* | 18 | - | 14 | 8.65 | 9.10 | 8.10 | 8.80 | - | *Gnpda1* | 5 | N.D. | N.D. | N.D. | N.D. | N.D. | N.D. | N.D. |
| MM117 | *Calm2* | 12 | - | 14 | 11.16 | 12.55 | 11.75 | 11.70 | - | *-* | 6 | N.D. | N.D. | N.D. | N.D. | N.D. | N.D. | N.D. |
| MM118 | *Ddx48* | 11 | - | 14 | 11.80 | 10.60 | 9.90 | 10.40 | - | *XP_485792.1* | 6 | N.D. | N.D. | N.D. | N.D. | N.D. | N.D. | N.D. |
| MM119 | *Pbp* | 5 | - | 14 | 12.90 | 13.75 | 13.60 | 13.80 | - | *XP_483886.1* | 6 | N.D. | N.D. | N.D. | N.D. | N.D. | N.D. | N.D. |
| MM120 | *Calm2* | 17 | - | 14 | 11.70 | 12.90 | 9.40 | 12.00 | MI | *-* | 6 | N.D. | N.D. | N.D. | N.D. | N.D. | N.D. | N.D. |
| MM121 | *Rbpsuh* | 5 | - | 14 | 9.86 | 6.80 | 6.45 | 7.45 | MI | *-* | 6 | N.D. | N.D. | N.D. | N.D. | N.D. | N.D. | N.D. |
| MM122 | *-* | 17 | - | 9 | 9.03 | 9.35 | 9.45 | 9.75 | - | *Tacstd2* | 6 | N.D. | N.D. | N.D. | N.D. | N.D. | N.D. | N.D. |
| MM123 | *Rap1a* | 3 | - | 14 | 10.41 | 6.50 | 7.20 | 8.10 | SO | *-* | 7 | N.D. | N.D. | N.D. | N.D. | N.D. | N.D. | N.D. |
| MM124 | *BC050188* | 8 | + | 0 | 3.75 | 6.16 | 8.60 | 7.50 | PM | *Trim61* | 8 | N.D. | N.D. | N.D. | N.D. | N.D. | N.D. | N.D. |
| MM125 | *Trim39* | 17 | - | 14 | 6.40 | 9.30 | 8.35 | 8.40 | ME | *Trim61* | 8 | N.D. | N.D. | N.D. | N.D. | N.D. | N.D. | N.D. |
| MM126 | *Rps15* | 10 | - | 14 | 13.10 | 11.95 | 11.25 | 11.95 | - | *-* | 8 | N.D. | N.D. | N.D. | N.D. | N.D. | N.D. | N.D. |
| MM127 | *2410127L17Rik* | 19 | - | 14 | 9.76 | 10.20 | 9.75 | 9.90 | - | *2410127L17Rik* | 9 | N.D. | N.D. | N.D. | N.D. | N.D. | N.D. | N.D. |

^1^ Names for parental genes and retrogenes were extracted from Ensembl annotation (field ‘display_id’ in main gene structure table).

^2^ Testis-specific expression is indicated with a ‘+’ sign. Rows with no determined expression are indicated (“N.D..”). Genes with expression but no testis specificity are labeled ‘-‘.

^3^ Number of somatic tissue among 14 where a gene is expressed.

^4^ SG, SC, ST and TT columns show the log 2 signal obtained for genes in spermatogonia, spermatocytes, spermatids, and total testis, respectively. Genes with no expression data are labeled (“N.D..”).

^5^ The specific stage where a gene is induced is indicated. SO, MI, ME, PM correspond to somatic, mitotic, meiotic and post-meiotic stages respectively. When a gene had no expression, its stage specificity was not determined (N.D..). Genes labeled “–” are expressed in one or more of the 4 spermatogenic stages, but no specific induction in any of these stages could be determined.
